# Homologous genes shared between probiotics and pathogens affect the adhesion of probiotics and exclusion of pathogens in the gut mucus of shrimp

**DOI:** 10.3389/fmicb.2023.1195137

**Published:** 2023-06-14

**Authors:** Yujie Sha, Qingyun Yan, Jian Liu, Jiafeng Yu, Shicai Xu, Zhili He, Jing Ren, Jie Qu, Shiying Zheng, Guomin Wang, Weiying Dong

**Affiliations:** ^1^Shandong Key Laboratory of Biophysics, Institute of Biophysics, Dezhou University, Dezhou, China; ^2^Shandong Engineering Laboratory of Swine Health Big Data and Intelligent Monitoring, Institute of Biophysics, Dezhou University, Dezhou, China; ^3^Southern Marine Science and Engineering Guangdong Laboratory (Zhuhai), Zhuhai, China

**Keywords:** *Lactiplantibacillus plantarum*, probiotics, mucus, adhesion, competitive exclusion

## Abstract

Clarifying mechanisms underlying the selective adhesion of probiotics and competitive exclusion of pathogens in the intestine is a central theme for shrimp health. Under experimental manipulation of probiotic strain (i.e., *Lactiplantibacillus plantarum* HC-2) adhesion to the shrimp mucus, this study tested the core hypothesis that homologous genes shared between probiotic and pathogen would affect the adhesion of probiotics and exclusion of pathogens by regulating the membrane proteins of probiotics. Results indicated that the reduction of FtsH protease activity, which significantly correlated with the increase of membrane proteins, could increase the adhesion ability of *L. plantarum* HC-2 to the mucus. These membrane proteins mainly involved in transport (glycine betaine/carnitine/choline ABC transporter *choS*, ABC transporter, ATP synthase subunit a *atpB*, amino acid permease) and regulation of cellular processes (histidine kinase). The genes encoding the membrane proteins were significantly (*p* < 0.05) up-regulated except those encoding ABC transporters and histidine kinases in *L. plantarum* HC-2 when co-cultured with *Vibrio parahaemolyticus* E1, indicating that these genes could help *L. plantarum* HC-2 to competitively exclude pathogens. Moreover, an arsenal of genes predicted to be involved in carbohydrate metabolism and bacteria-host interactions were identified in *L. plantarum* HC-2, indicating a clear strain adaption to host’s gastrointestinal tract. This study advances our mechanistic understanding of the selective adhesion of probiotics and competitive exclusion of pathogens in the intestine, and has important implications for screening and applying new probiotics for maintaining gut stability and host health.

## 1. Introduction

Probiotics are defined as active microorganisms that are beneficial to the host, they can confer several benefits such as improving growth, digestion, and immunity (Parker, 1974; Zhang et al., 2018). Probiotics are widely used as alternatives to antibiotics. For example, *Lactobacillus* (e.g., *L. casei*, *L. gasseri*, *L. helveticus*, *L. lactis*, *L. rhamnosus*, *L. reuteri*, and *L. acidophilus*) and *Lactiplantibacillus* (*L. plantarum* and *L. pentosus*) have health-promoting effects (Kankainen et al., 2009; Zhang et al., 2018; Partrick et al., 2021; Wang et al., 2021), and are commonly accepted probiotics strains in the market.

Adhesion to the gut is important to the colonization of probiotics, which is conducive to the proliferation of probiotics but harmful to the pathogens in the intestine (Reid and Burton, 2002; Chen et al., 2007; Yadav et al., 2017). The molecules such as surface proteins, cell wall polysaccharides, peptidoglycans, bacteriocins, secreted proteins, and organic acids derived from probiotics *Lactobacilli* can mediate bacterial adhesion (Teame et al., 2020). The surface protein is one of the major adhesion molecules involved in probiotic mechanisms among different taxonomic groups of probiotics (Sanders et al., 2018). For example, *L. casei* surface layer protein could reduce the adhesion of pathogens to collagen and HT-29 cells, and thus decrease the pathogens-induced apoptosis of HT-29 cells (Meng et al., 2021). *L. acidophilus* surface layer protein also presented antibacterial effects against *Staphylococcus aureus* in combination with nisin (Wang et al., 2020).

Results from our previous studies suggested that the molecules located on the surface of *L. plantarum* HC-2 might participate in the competitive exclusion of *Vibrio parahaemolyticus* (Sha et al., 2016a), and the surface layer proteins of *L. plantarum* HC-2 were subsequently found to play an important role in its adhesion and colonization in the intestine of shrimp to competitively exclude the pathogens and promote beneficial bacteria growth (Du et al., 2019). However, whether other genes or proteins of *L. plantarum* HC-2 are involved in the adhesion and exclusion of pathogens remains unclear. Probiotics and pathogens compete for common cell receptors for binding to the intestine mucosa (Van den Abbeele et al., 2016; Singh et al., 2018). Thus, understanding the surface proteins of probiotics involved in host-probiotic interaction would provide new strategies for disrupting host-pathogen interaction.

Moreover, *L. plantarum* HC-2 could reduce the negative effects of aflatoxin by positively affecting the growth and resistance of *P. vannamei* (Fang et al., 2020). Thus, some other modes of action may exist for *L. plantarum* HC-2 to find a niche in the shrimp intestine. Different bacterial species may have unique biological activities, which may vary from one strain to another strain. So, the unique property is fundamental to understand and apply a specific probiotic strain (Minj et al., 2021). The characteristics of the genome and proteome could reflect the adaptability and function of the bacteria to specific niches, so that the probiotic properties of *L. plantarum* HC-2 can be predicted by analyzing its whole genome and proteome (O'sullivan et al., 2009; De Angelis et al., 2016).

This study mainly aimed to investigate which and how the genes and proteins of *L. plantarum* HC-2 affect the adhesion and exclusion of pathogens. We hypothesized that (i) the homologous genes shared between *L. plantarum* HC-2 and *V. parahaemolyticus* would affect the adhesion of *L. plantarum* HC-2 and competitive exclusion against pathogens in the mucus of shrimp by regulating the quantity of membrane proteins; (ii) the genome of *L. plantarum* HC-2 harbored key genes contributing to its adaptability to shrimp intestine niche. So, we first tested whether the shared genes between *L. plantarum* HC-2 and *V. parahaemolyticus* participated in adhesion of *L. plantarum* HC-2 to mucus, and examined the proteins involved in the adhesion of *L. plantarum* HC-2 to mucus and the response to *V. parahaemolyticus* exposure. Secondly, we analyzed the other probiotic characteristics of *L. plantarum* HC-2 based on its genome. We found that *ftsH* gene could affect the adhesion of *L. plantarum* HC-2 by regulating several membrane proteins, some of which play important roles in the competitive exclusion of pathogens. Arsenal genes in *L. plantarum* HC-2 genome are predicted to be involved in carbohydrate metabolism and bacteria-host interactions. These findings advance our mechanistic understanding of the selective adhesion of probiotics and competitive exclusion of pathogens in the intestine of shrimp, and have an important practical significance for the screening of probiotics, the optimization of feed additive formulations and the defense against diseases.

## 2. Materials and methods

### 2.1. Bacterial strains and culture conditions

*Lactiplantibacillus plantarum* HC-2 was isolated from the hindgut of fish (*Acanthogobius hasta*) and stored at −80°C in Man, Rogosa, and Sharpe (MRS) broth (Qingdao Hope Biol-Technology Co., Ltd., Qingdao, China) containing 20% (v/v) glycerol. *V. parahaemolyticus* E1 was stored at −80°C in 2216E broth (Qingdao Hope Biol-Technology Co., Ltd., Qingdao, China) containing 20% (v/v) glycerol. *L. plantarum* HC-2 was cultured at 37°C under anaerobic conditions in MRS broth for 18 h, and *V. parahaemolyticus* E1 was cultured aerobically in 2216E broth at 28°C for 18 h as seed culture.

### 2.2. Genome sequencing and annotation

*Lactiplantibacillus plantarum* HC-2 was collected by centrifugation (12,000 g) at 4°C for 10 min. The DNA was extracted with a bacterial genomic DNA extraction kit (Tiangen, China) according to the manufacturer’s instructions. The quality of DNA was verified by 1% (w/v) agarose gel electrophoresis and quantified by Qubit^®^ 2.0 Fluorometer (Thermo Scientific). The whole genome was sequenced by PacBio Sequel platform and Illumina NovaSeq PE150 at the Beijing Novogene Bioinformatics Technology Co., Ltd. Genomes were assembled using SMRT Link (v5.0.1). The low-quality reads (< 500 bp) were filtered, and the long reads (> 6,000 bp) were chosen as seed sequences. The sequences ranged from 500 to 6,000 bp were aligned to the seed sequence by Blasr to improve the accuracy. The obtained sequences were then corrected by the arrow algorithm in the variant Caller module of the SMRT Link software, which was then used as reference sequences to blast with Illumina data by bwa (filtered with base minimum mass value of 20, read depth of 4–1,000). The cyclization of chromosomal sequence depending on the overlap between the head and the tail, and the initial site was then corrected by blast with the DNA database. Finally, the plasmid database was used to blast the chromosome and plasmid sequences. The coding genes, transfer RNA genes, and ribosome RNA genes were predicted by GeneMarkSprogram, tRNAscan-SE, and rRNAmmer, respectively. Gene ontology (GO), Cluster of Orthologous Groups (COG) and Kyoto Encyclopedia of Genes and Genomes (KEGG) were used to predict the function of genes by blasting the whole genome (E-value of 1 × 10–5, minimal alignment length percentage of 40%).

### 2.3. Multiple alignments of conserved genomic sequences with Mauve

A total of 29 genomes of *V. parahaemolyticus* were found at the 100% assembly level as of December 2018 from the National Center for Biotechnology Information (NCBI) genome database (the identifier for the genome assembly can be found in Supplementary Table S1). The multiple alignments of genomes between *L. plantarum* HC-2 and *V. parahaemolyticus* strains was performed using progressive Mauve option in software Mauve (Aaron et al., 2004). The consensus sequences existed in *L. plantarum* HC-2 and all *V. parahaemolyticus* strains were used as conserved genomic sequences for subsequent analysis.

### 2.4. Deletion of the *ftsH* gene

A double-crossover mutagenesis was performed to construct the in-frame deletion of *ftsH*. In brief, two fragments (about 1 Kb) flanking *ftsH* were amplified independently with primers 464up-F-XhoI and 464up-R, or 464do-F and 464do-R-EcoRI. The obtained fragments were joined together by overlap PCR using primers 464do-F and 464up-R. The resulting fusion fragment was introduced into the temperature sensitive plasmid pG^+^host9 (Okano et al., 2009) through XhoI and EcoRI. The constructed plasmid was amplified in *E. coli* DH5α and transformed into *L. plantarum* HC-2 by electroporation. The transformants were selected by erythromycin (300 μg/mL) at 30°C.

The transformants were then separated as single clones at 37°C overnight. The integration of the plasmid to the genome was verified by primers Test464-F2 and Test464-R2 product length 1 Kb or 3 Kb. The integrated clones were inoculated in medium without erythromycin for 7–10 generations and spread as single clones on non-selective plates. The erythromycin resistance of these clones was confirmed through inoculation on plate with or without erythromycin (300 μg/mL). Clones loosing erythromycin resistance were checked by primers Test464-F2 and Test464-R2 and the resulting length 1 Kb indicated Δ*ftsH*. The specific primers used in present study were listed in Supplementary Table S2.

### 2.5. Adhesion ability of strains to shrimp mucus

The preparation of shrimp mucus and adherence experiments were performed as previously study (Sha et al., 2016b). In briefly, the experimental shrimp were obtained after being fed for 4 h from Ruizi Seafood Development Co.Ltd. (Qingdao, China). Crude mucus was obtained from the intestines of shrimp by gentle scraping and was suspended in cold HEPES-Hanks’s Buffer (H-H Buffer). Then, the solution was centrifuged to remove non-soluble material and was diluted with H-H Buffer to a final concentration of 1 mg/mL. 100 μL prepared shrimp mucus was added into the wells of a 96-well micro-titer plate and incubated overnight at 4°C. Then, the wells were washed three times with sterile PBS to remove the non-immobilized mucus. Subsequently, the Δ*ftsH* mutant, Δ*ftsH*-C and wild type bacteria were added to the immobilized mucus and incubated at 37°C for 1 h, respectively. Wells were washed five times with sterile PBS and treated with 0.05% trypsin (100 μL) for 10 min at room temperature to liberate the bacteria. The liberated bacteria were then serially diluted (10^3^ ~ 10^5^) and plated on MRS plates, which incubated at 37°C under anaerobic conditions for 24 h. The number of colony-forming units (CFUs) was counted, and the adhesion ability of each type strain was calculated by normalizing the liberated bacteria CFUs to total CFUs before adhesion.

### 2.6. Sample preparation for proteomic analysis, liquid chromatography, and mass spectrometry

Cells from *L. plantarum* HC-2 wild type and mutant were collected at the stationary phase for differential proteome analysis. The bacterial pellets were collected by centrifugation (12,000 *g*) at 4°C for 10 min, and then transferred to 1.5 mL centrifuge tube with SDT lysis buffer (4% SDS, 10 mM DL-DTT, 100 mM TEAB) before ultrasonication for 5 min on ice. The lysate was centrifuged at 12,000 *g* for 15 min at 4°C for collecting the supernatant, which was then reduced with 10 mM DTT for 1 h at 56°C. Sufficient iodoacetamide was added to alkylate for 1 h at room temperature in the dark. Subsequently, four volumes pre-cold acetone were added and mixed completely, and the mixture were incubated at −20°C overnight. The protein precipitation was washed with 1 mL pre-cold acetone and then dissolved in dissolution buffer (8 M Urea, 100 mM TEAB, pH = 8.5). Protein concentration of the lysate was determined by Bradford method (Bradford, 1976). For each sample, 20 μg proteins were loaded on the 12% SDS-PAGE. After a concentration with 80 V for 20 min, the protein was separated by 120 V for 90 min, and then visualized using coomassie brilliant blue R-250. The trypsin treatment and desalted were performed as previous study (Zhang et al., 2016).

Peptides were analyzed using an EASY-nLC™ 1,200 nano UHPLC system connected to a Q Exactive™ HF-X mass spectrometer (Thermo Fisher) with ion source of Nanospray Flex™ (ESI). In data dependent mode, the mass spectrometer automatically switched between MS1 and MS2 spectra. MS1 spectra had a full scan mass-to-charge (*m*/*z*) range from 350 to 1,500 using a maximum injection time of 20 ms and an automatic gain control (AGC) target value of 3 × 10^6^. Top 40 abundant precursors were isolated, fragmented by higher energy collisional dissociation (HCD) using 27% normalized collision energy (NCE) and analyzed at a resolution of 15,000 with a scan range from 200 to 2,000 m/z with an AGC target value of 1 × 10^5^. The raw data of MS detection was acquired by set the intensity threshold of 2.2 × 10^4^ and the dynamic exclusion parameter of 20 s.

### 2.7. Protein identification and quantification

Peptide/protein identification and quantification were performed with Proteome Discover 2.2 (PD2.2, Thermo) by searching the raw data against database consisting of 22,110 sequences obtained from the zip file named Match_result-X101SC21061420_Z01_J001_B1_43 in ProteomeXchange Consortium with the dataset identifier PXD036035. The search parameters were as follows: mass tolerance for precursor ion and product ion was 10 ppm and 0.02 Da, respectively; fixed modification with carbamidomethyl; dynamic modification with methionine oxidation; N-Terminal modification with acetylation; allowance of 2 missed cleavage; identified peptides spectrum matches with a credibility of more than 99% and at least one unique peptide; false discovery rate set as 0.01. The mutant strains with fold changes ≥1.5 or ≤ 0.67, and *p* value ≤0.05 (*t*-test) were considered as significantly differentially expressed proteins.

### 2.8. Bioinformatics analysis of protein

GO and InterPro (IPR) functional analysis were performed by the interproscan software against the non-redundant database (including Pfam, PRINTS, ProDom, SMART, ProSite, PANTHER). The protein family and pathway were analyzed by COG and KEGG. Subcellular localization for each protein was predicted according to GO annotation by UniProt.^1^ The protein–protein interactions were predicted using the STRING-DB software.^2^

### 2.9. Expression of adhesion-related genes in *Lactiplantibacillus plantarum* HC-2 cocultured with *Vibrio parahaemolyticus* E1

Establishment of the mono-and co-culture system for *L. plantarum* HC-2 and *V. parahaemolyticus* E1 were performed as described previously (Sha et al., 2016a). In briefly, equal volumes of MRS and 2216E broth were mixed, and 150 mL of mixed medium was inoculated with 150 μL of the *L. plantarum* HC-2 and *V. parahaemolyticus* E1 seed cultures and incubated at 28°C for 36 h. Monocultures of both strains were prepared as a control. Samples were collected to monitor the adhesion-related gene expression at *t* = 3, 6, 9, 12, 24, and 36 h, respectively. After the samples were withdrawn, an equal volume of the mixed medium was added to ensure that the volume of the cultures remained at 150 mL.

The methods of RNA isolation, reverse transcription, and quantitative real-time PCR (qPCR) were performed according to previous study (Sha et al., 2016b). In briefly, RNA isolation was performed from bacteria using an RNA fast extraction kit according to the manufacturer’s protocol (Fastagen, China). 2 μg of RNA was used for reverse transcription using a TransScript^®^ One-Step gDNA Removal and cDNA Synthesis Kit according to the manufacturer’s protocol (TransGen Biotech Co., Ltd., China). qPCR was performed using TransStar Top Green qPCR Supermix according to the manufacturer’s protocol (TransGen Biotech Co., Ltd., China) with three replicates of each sample under the following two steps: denaturation at 94°C for 30 s and then 40 cycles of 94°C for 5 s and 60°C for30 s. The specificity of PCR products were confirmed by the dissociation curve analysis at the end of qPCR. Microsoft Excel was used to analyze the data and the expression changes were determined with the 2^−ΔΔCt^ method. *MA1* and *pvuA* were selected as the reference genes for *L. plantarum* HC-2 and *V. parahaemolyticus* E1, respectively. Specific primers used in present study were listed in Supplementary Table S2.

### 2.10. Statistical analysis

All statistical analyses were performed with SPSS (version 20), and student’s *t*-test was used to analyze differences between two compared groups with a significant level at *p* ≤ 0.05. PCA analysis was performed with the R package vegan. The graphic works were performed with origin 8.0 or R software.

### 2.11. Data availability

The raw sequencing data and assembly sequence of *L. plantarum* HC-2 genome can be found at the National Center for Biotechnology Information (NCBI) (BioProject: PRJNA868485; Sequence Read Archive (SRA): SRR21010423). The mass spectrometry proteomics data of *L. plantarum* HC-2 have been deposited to the ProteomeXchange Consortium *via* the PRIDE (PRoteomics IDEntification Database) partner repository with the dataset identifier PXD036035.

## 3. Results

### 3.1. General genome features of a probiotic *Lactiplantibacillus plantarum* HC-2

The complete genome of *L. pentousus* HC-2 consists of 3,364,894 nucleotides (one chromosome and two plasmids) with an average GC content of 45.41%. *In silico* analyses revealed the presence of 3,259 open reading frames (ORFs), resulting in a coding percentage of 84.12%. One or more protein families (PFAM) are attributed to 66.68% of these ORFs; 72.72% of them have similarities (> 97%) to at least one COG; 1.17% of the ORFs remain unknown and 79.78% of them show similarities (> 97%) to defined genes.

All of these circles are fairly symmetrical in the genome by observing the predicted origin of replication through GC-skew analysis and the ORF orientation shift (Supplementary Figure S1, circles 2 to 5 and 8). The five rRNA loci are located in the GC-content spikes (average GC content of 52.66%) (Supplementary Figure S1, circle 6) and all rRNA loci are in the same direction as the DNA replication (Supplementary Figure S1, circle 7). Sixty-four tRNAs are identified in the genome, representing all 20 amino acids, with redundant tRNAs for all amino acids except tryptophan and cysteine.

### 3.2. Conservation of genome architecture between *Lactiplantibacillus plantarum* HC-2 and *Vibrio parahaemolyticus*

Nearly 20,000 sequences were obtained which were homologous between *L. plantarum* HC-2 and at least one of *V. parahaemolyticus* strains. In order to ensure that the sequences were conserved in both *L. plantarum* HC-2 and *V. parahaemolyticus* strains, the presence of homologous sequences in all studies strains was selected as the filter. Seventeen sequences were filtered out which were homologous between *L. plantarum* HC-2 and all *V. parahaemolyticus* strains, and then were assigned to 11 genes based on their location in *L. plantarum* HC-2 genome (Table 1). These genes mainly attribute to the essential cellular metabolic processes such as transcription, translation, glycolysis, and protein stability (Table 1).

**Table 1:** Eleven genes of *Lactiplantibacillus plantarum* HC-2 with homology to all investigated *Vibrio parahaemolyticus* genomes.

Gene ID	Locus	Putative gene	Description	Homologous region coverage	Organism	E value	Per. Ident.	Accession NO. (NCBI)
HC2_GM001536	1,596,833:1598839	*parE*	DNA topoisomerase IV subunit B	82.71%	*L. plantarum*	3e-38	100%	WP_003640557.1
HC2_GM001644	1,714,477:1715376	*glyQ*	Glycine--tRNA ligase subunit alpha	100%	*L. plantarum*	0	99.64%	WP_076638239.1
HC2_GM000845	893,611:894147	*rplF*	50S ribosomal protein L6	54.56%	*L. plantarum*	2e-63	100%	WP_114668113.1
HC2_GM000571	603,940:606303	*secA*	Protein export cytoplasm protein SecA ATPaseRNA helicase	52.96%	*L. plantarum*	1e-141	100%	KZU90851.1
HC2_GM001758	1,832,001:1833266	*clpX*	ATP-dependent protease	33.81%	*L. plantarum*	1e-95	100%	KLD58150.1
HC2_GM000995	1,050,186:1051763	*prfC*	Peptide chain release factor 3	48.42%	*L. plantarum*	4e-112	100%	SPX97467.1
HC2_GM001692	1,763,037:1765613	*infB*	Translation initiation factor IF-2	56.30%	*L. plantarum*	0	100%	AVV99227.1
HC2_GM000464	486,732:488984	*ftsH*	ATP-dependent zinc metalloprotease FtsH	45.23%	*L. plantarum*	0	100%	WP_064775395.1
HC2_GM000625	664,914:666242	*eno*	Phosphopyruvate hydratase	90.29%	*L. plantarum*	0	100%	WP_134992604.1
HC2_GM000828	883,533:885629	*fusA*	MULTISPECIES: elongation factor G	100%	*Lactobacillus*	0	100%	WP_003641250.1
HC2_GM000826	882,491:882904	*rpsL*	30S ribosomal protein S12	30.43%	*L. plantarum*	1e-20	100%	WP_080453582.1

Eleven genes are all located on the chromosome.

### 3.3. The role of *ftsH* gene and related proteins in the adhesion of *Lactiplantibacillus plantarum* HC-2 to the mucus of shrimp

**Figure 1:**
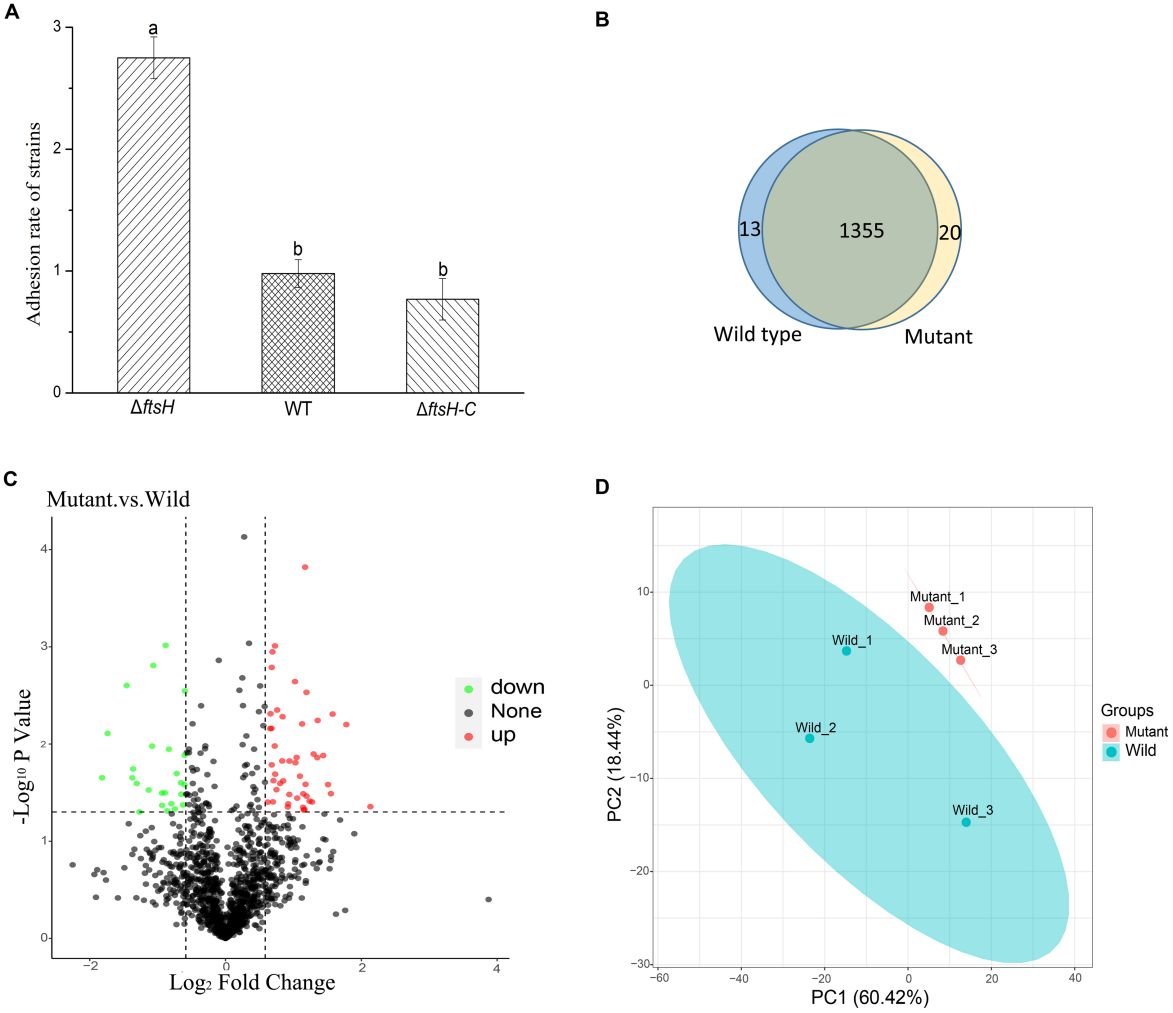
The adhesion of *Lactiplantibacillus plantarum* HC-2 is affected by FtsH expression. **(A)** The adhesion rate of wild type (WT), *ftsH* mutant (Δ*ftsH*), and *ftsH* mutant complemented with the *ftsH* (Δ*ftsH*-C). **(B)** The Veen diagram shows the unique and shared proteins between wild type and mutant groups. **(C)** Volcano plot of changes at the protein level of *L. plantarum* HC-2 after FtsH expression decreased. **(D)** Principal coordinates analysis based on the Unifrac distance.

In order to elucidate the role of *ftsH* in the adhesion of *L. plantarum* HC-2 to shrimp mucus, a mutant strain was constructed by double-crossover mutagenesis. PCR results confirmed the absence of *ftsH* in *L. plantarum* HC-2 genome (data not shown). One mutant was screened out from nearly 5,000 mutants. The Δ*ftsH* mutant exhibited a significantly (*p* < 0.05) higher adhesion rate than the wild type (more than 2 times) (Figure 1A). When complemented with the *ftsH*, the adhesion rate nearly restored the level of the wild type (Figure 1A). The results provide evidence that a strong connection between the adhesion ability and FtsH role does exist in *L. plantarum*.

**Figure 2:**
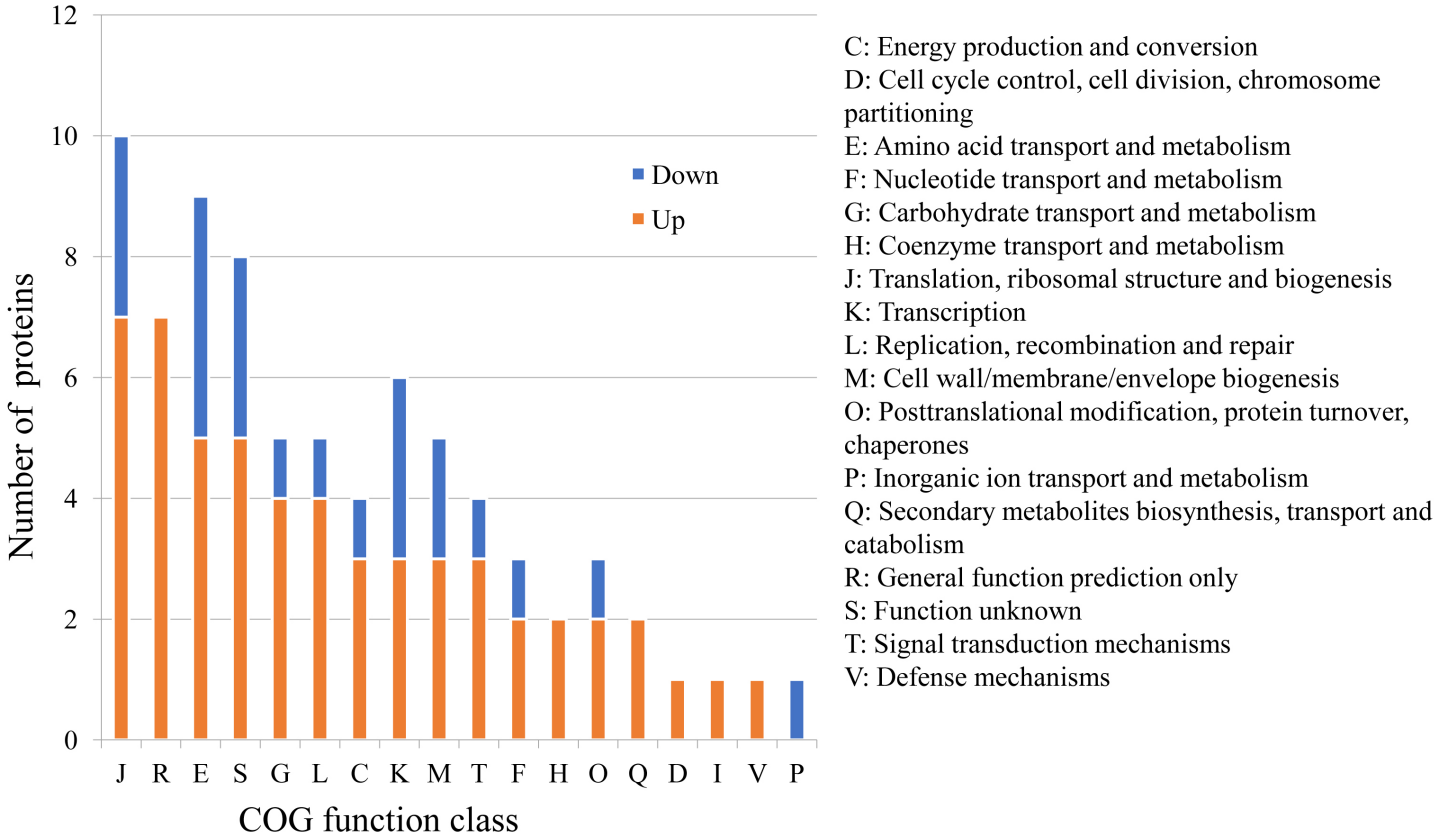
Predicted functions of differentially expressed proteins between wild type and mutant strains based on the Cluster of Orthologous Groups (COG) analysis.

Because of the regulatory protease activity of FtsH (Bove et al., 2012), we sought to ascertain whether inactivation of *ftsH* could alter the surface proteins of *L. plantarum*. A total of 1,369 and 1,376 proteins were detected in the wild type and mutant, respectively (Figure 1B). In addition, we identified that 26 proteins were significantly down-expressed and 54 proteins were significantly up-expressed (Figure 1C). Although the deletion of *ftsH* inhibited the expression of several genes, the expression of more proteins was up-regulated. The up-regulated proteins mainly belonged to the following categories: (i) translation, ribosomal structure and biogenesis, (ii) general function, (iii) amino acid transport and metabolism, (iv) carbohydrate transport and metabolism, and (v) replication, recombination and repair (Figure 2). The down-regulated proteins were also involved in mostly of the functions indicated above, but the numbers were less than the up-regulated proteins. Global proteomic profiles of the wild type and mutant strains were compared revealing a distinct separation between these two types of strains, suggesting that *ftsH* deletion induced the expression changes of proteins which may contribute to the increased adhesion ability of *L. plantarum* HC-2 (Figure 1D). Among the differentially expressed proteins, six proteins were considered to be related to the adhesion of *L. plantarum* HC-2 due to their higher expression in the membrane of mutant strains (Table 2) and were selected as adhesion-related biomakers.

**Table 2:** Differentially expressed proteins predicted to be located on the cell membrane.

Protein ID (Uniprot)	Description	GO_Term	GO_Class	Gene	E_value	Up vs. Down
G0M1M7	Cell division inhibitor MinD	--	--		1e-138	up
I8R6Y9	Oligopeptide ABC transporter, substrate binding protein	--	--	*oppA1*	7e-28	down
I9L3P8	Glycine betaine/carnitine/choline ABC transporter, substrate binding and permease protein	Transporter activity	MF	*choS*	7e-39	up
I9AKC3	ABC transporter, ATP-binding and permease protein	Transport, transmembrane transport, catalytic activity, membrane	BP, BP, MF, CC		6e-59	up
A0A2S9W1Y1	ATP synthase subunit a	Single-organism transport, ribose phosphate metabolic process, hydrogen ion transmembrane transporter activity, ATP synthesis coupled proton transport, nucleotide metabolic process, single-organism process, nucleobase-containing compound metabolic process, transporter activity, transport, substrate-specific transmembrane transporter activity, nucleoside monophosphate metabolic process, nucleoside phosphate metabolic process, transmembrane transport	BP, BP, MF, BP, BP, BP, BP, MF, BP, MF, BP, BP, BP	*atpB*	3e-20	up
F6IZF7	Histidine kinase	Regulation of cellular process, phosphotransferase activity, alcohol group as acceptor, transferase activity, phosphorelay sensor kinase activity, cellular response to stimulus, transferase activity, transferring phosphorus-containing groups, signal transduction, single-organism process, catalytic activity	BP, MF, MF, MF, BP, MF, BP, BP, MF		2e-26	up
F6ISB0	Glycine betaine/carnitine/choline ABC transporter, ATP-binding protein	Transport, membrane	BF, CC		1e-106	down
A0A241RSP9	Amino acid permease	Membrane, transport, transmembrane transport	CC, BP, BP		7e-87	up

### 3.4. The responses of potential adhesion-related biomarkers to *Vibrio parahaemolyticus* E1

**Figure 3:**
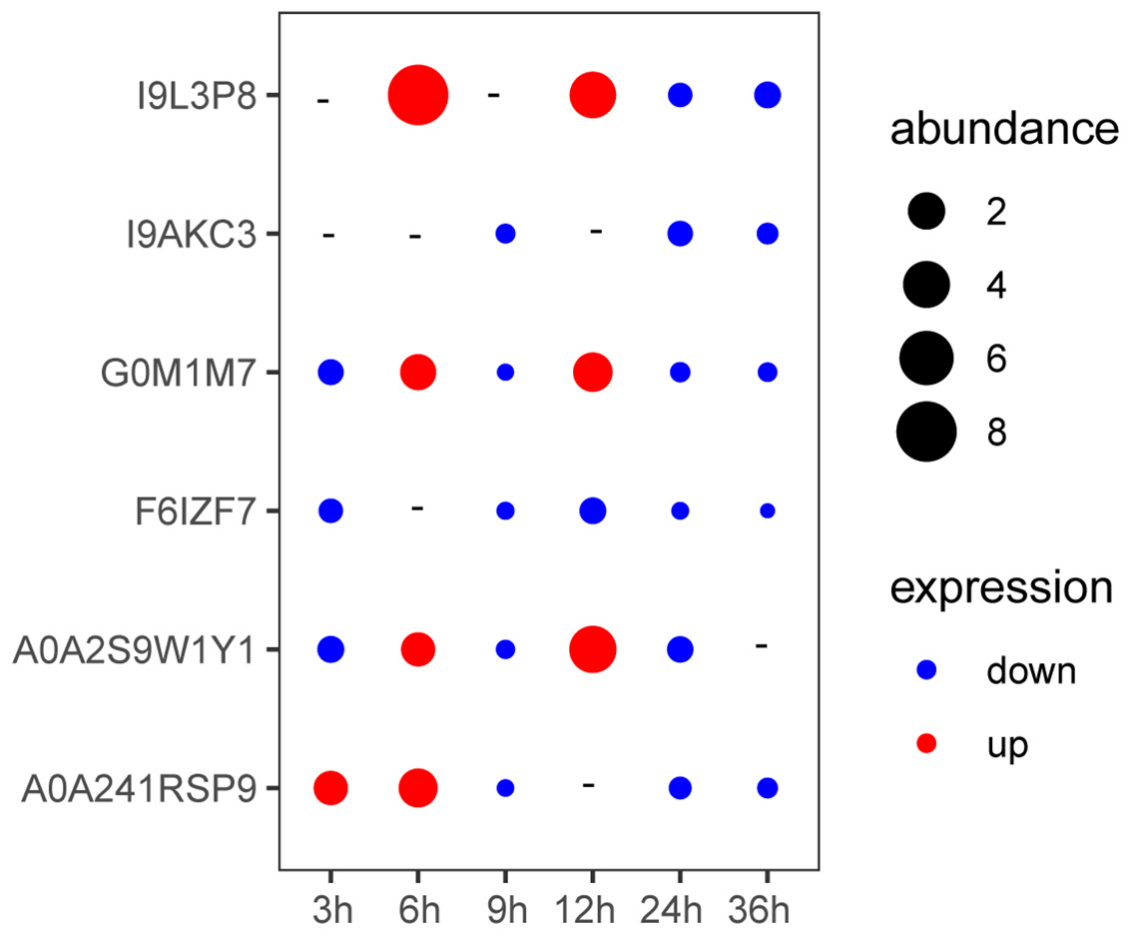
The expression of genes encoding membrane proteins in *Lactiplantibacillus plantarum* HC-2 when co-cultured with *Vibrio parahaemolyticus* E1 at different time points. “-” means no change. “abundance” means the expression fold-changes of the target gene relative to the reference gene.

As shown in Figure 3, the expression of genes coding A0A241RSP9 was significantly up-regulated at the presence of *V. parahaemolyticus* E1 for 3 h. The expression of genes coding G0M1M7, I9L3P8, A0A2S9W1Y1, and A0A241RSP9 were significantly up-regulated when co-cultured with *V. parahaemolyticus* E1 for 6 h. However, the expression of gene coding I9L3P8 was 8-fold higher than that of the control group. At 12 h, three membrane protein-coding genes (I9L3P8, G0M1M7, A0A2S9W1Y1) were up-regulated, and the expression of I9L3P8 and A0A2S9W1Y1 were 4-fold higher than that of the control group. Thus, the presence of pathogenic bacteria can induce the expression of *L. plantarum* HC-2 surface-associated proteins.

### 3.5. Adaption mechanisms of *Lactiplantibacillus plantarum* HC-2 to the host intestinal environment and interaction with the host

*Lactiplantibacillus plantarum* HC-2 has been shown to be able to ferment *in vitro* a variety of carbohydrates (Sha, 2016). Among them, Methyl-α-D-Mannopyranoside, N-acetylglucosamine, amygdalin, Arbutin, Esculin ferric citrate, salicin, D-lactose, D-melibiose and D-melezitose could be utilized by *L. plantarum* HC-2 as the only carbon source, but the related genes were not found in the genome of *L. plantarum* HC-2, which need to be further investigated. In addition, *in silico* analysis of the annotated genome sequence of *L. plantarum* HC-2 also predicted its capacity to ferment mannitol, inositol and glycogen.

**Figure 4:**
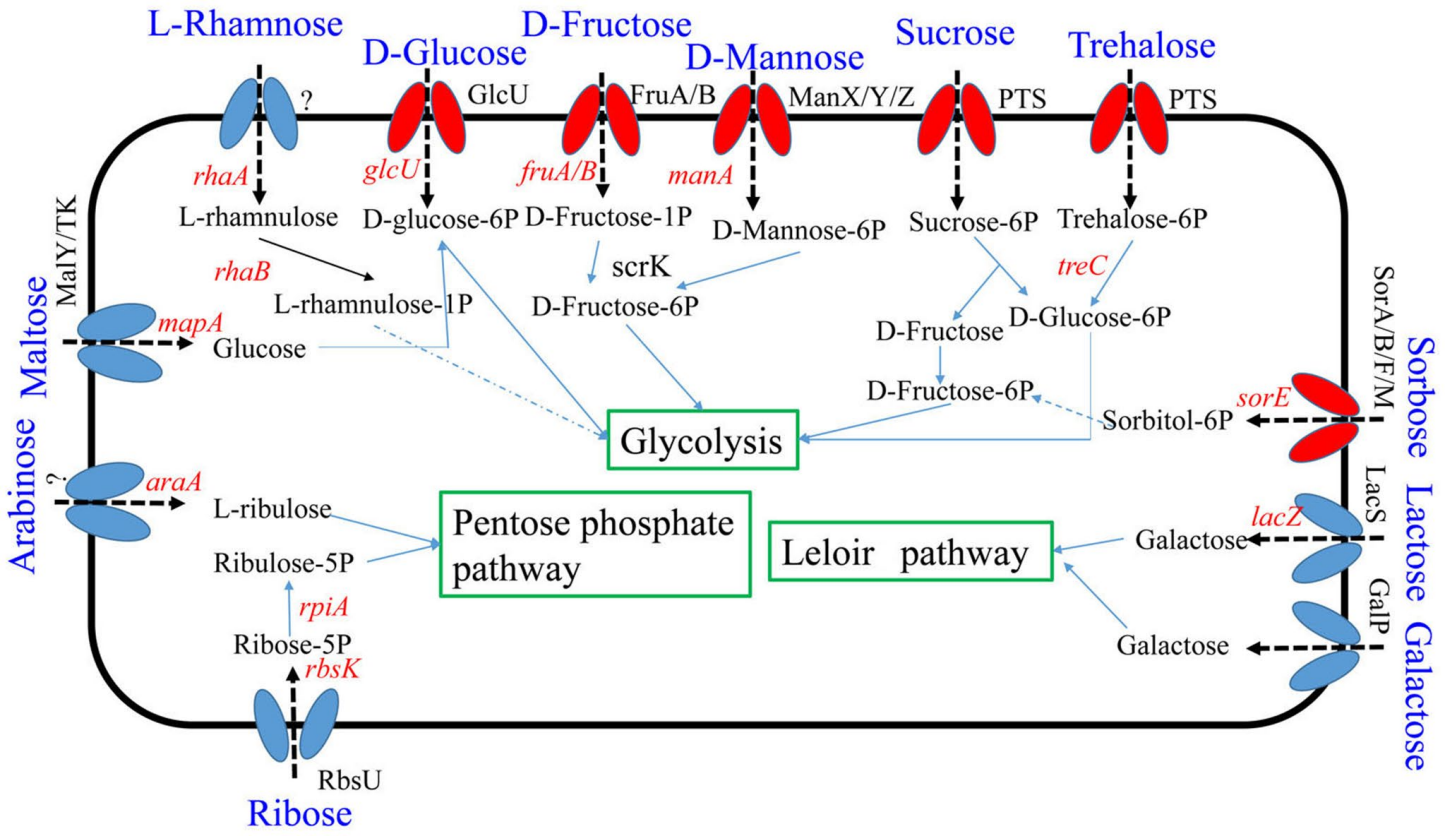
Predicted carbohydrate metabolism pathways of *Lactiplantibacillus plantarum* HC-2. Red: phosphotransferase system; blue: permease protein.

**Figure 5:**
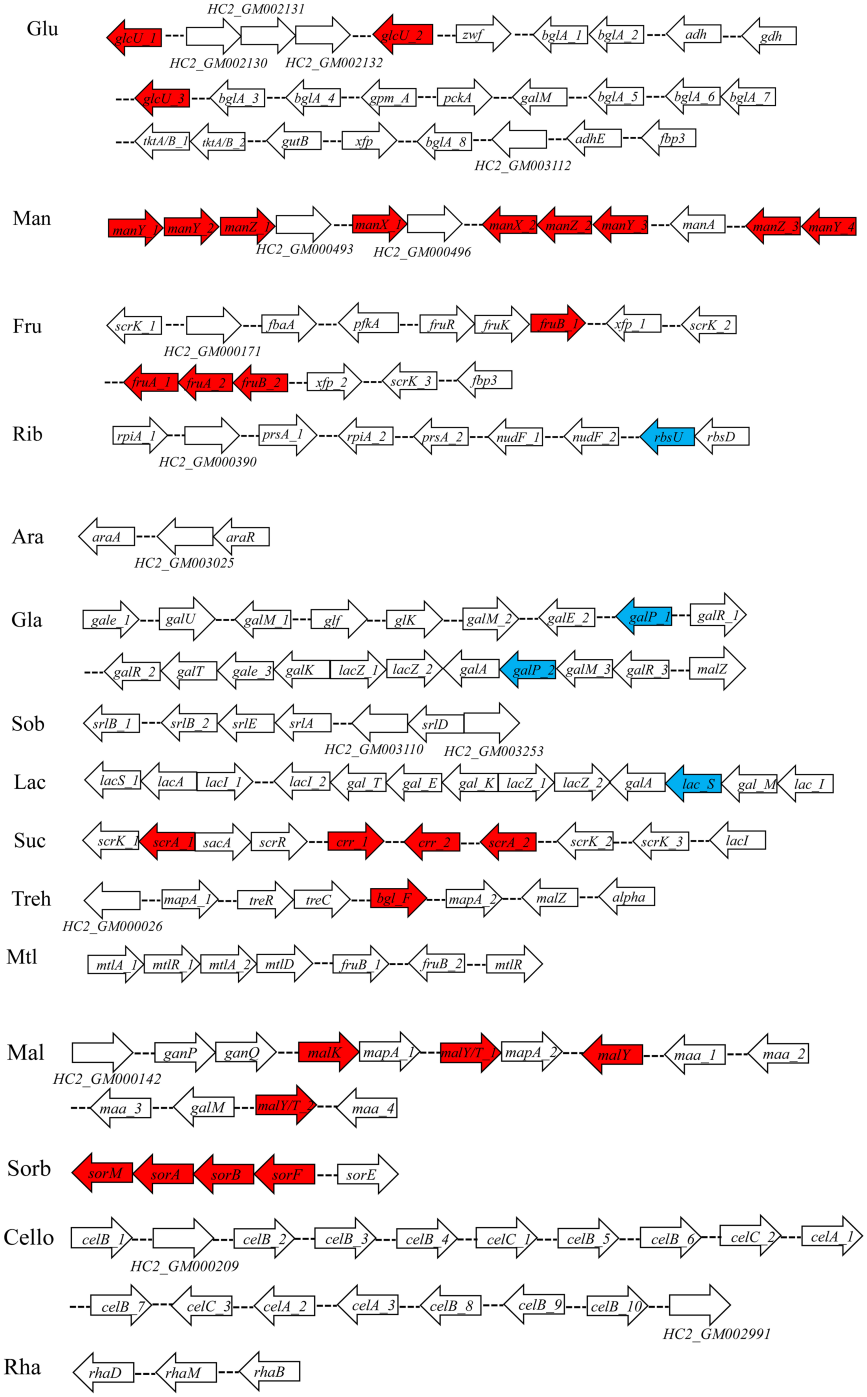
Predicted gene clusters involved in major carbohydrate metabolism. Red: phosphotransferase system; blue: permease protein. Glu, glucose; Man, mannose; Fru, fructose; Rib, ribose; Ara, arabinose; Gla, galactose; Sob, sorbitol; Lac, lactose; Suc, sucrose; Treh, treholose; Mtl, mannitol; Mal, maltose; Sorb, Sorbose; Cello, cellobiose; Rha, rhamnose.

Seven percent of the identified genes in the *L. plantarum* HC-2 genome were involved in carbohydrate metabolism. The analysis of BlastKOALA predicted that *L. plantarum* HC-2 possessed three complete carbohydrate metabolism pathways involving EMP, pentose phosphate pathway and galactose metabolism (Figure 4), these pathways formed the central core of carbohydrate metabolism of *L. plantarum* HC-2. In addition, 15 carbohydrate utilization pathways were predicted in the genome of *L. plantarum* HC-2. As such, the wide repertoire of enzymes involved in carbohydrate metabolism are found in its genome, which are also corroborated by the abundant number of genes for the phosphoenolpyruvate- (PEP) dependent sugar phosphotransferase system (PTS) (67 genes) and the presence of specific genes or gene clusters involved in carbohydrate utilization by *L. plantarum* HC-2 (Figure 5).

The adhesion genes of probiotic *L. plantarum* HC-2 were also analyzed, combined with annotation and published studies (Van Tassell and Miller, 2011; Abriouel et al., 2017). A total of 34 adhesion genes were predicted in the genome of *L. plantarum* HC-2 (Table 3).

**Table 3:** Predicted genes encoding proteins with roles in adhesion or interaction with the host in the genome of *Lactiplantibacillus plantarum* HC-2.

Gene ID	Locus	Description	Species	Query cover	E value	Per. Ident.	Accession NO. (NCBI)
HC2_GM000186	203,881:204351	Malate/L-lactate/L-2-hydroxyisocapronate dehydrogenase	*L. plantarum* ZJ316	99%	0	100%	AGE39687.1
HC2_GM000189	205,446:206159	Phosphate acetyltransferase	*Lactobacillaceae*	99%	0	100%	WP_003641060.1
HC2_GM000284	301,660:302523	Elongation factor Tu	Bacteria	99%	0	100%	WP_003640798.1
HC2_GM000304	323,033:323596	Cell surface protein	*Lactobacillaceae*	99%	3.00E-67	100%	WP_003641618.1
HC2_GM000365	379,701:380246	Membrane protein	*L. plantarum*	99%	8.00E-97	100%	GFH83498.1
HC2_GM000560	591,941:592225	Phosphoglycerate mutase	*L. plantarum* JDM1	99%	1.00E-163	100%	ACT63123.1
HC2_GM000561	592,281:593897	Molecular chaperone DnaK	*L. plantarum*	98%	0	99.67%	WP_160230933.1
HC2_GM000624	664,107:664832	Co-chaperone GroES	*Lactobacillaceae*	98%	2.00E-50	98.94%	WP_003640985.1
HC2_GM000639	687,863:688840	F0F1 ATP synthase subunit beta	*Lactobacillaceae*	99%	0	100%	WP_003641443.1
HC2_GM000799	847,568:847768	Integral membrane protein	*L. plantarum* ST-III	98%	1.00E-52	98.82%	ADN98013.1
HC2_GM001027	1,088,208:1089281	Integral membrane protein	*L. plantarum*	99%	0	100%	WP_022638051.1
HC2_GM001143	1,220,958:1221632	Membrane-associated phospholipid phosphatase	*L. plantarum*	99%	2.00E-140	100%	KRN36053.1
HC2_GM001291	1,359,172:1359654	Class II fructose-1,6-bisphosphate aldolase	*Lactobacillaceae*	99%	0	100%	WP_003641904.1
HC2_GM001415	1,475,138:1475353	Integral membrane protein	*L. plantarum* JDM1	98%	3.00E-15	96.97%	ACT62313.1
HC2_GM001425	1,481,769:1482131	Integral membrane protein	*L. plantarum* JDM1	98%	7.00E-47	100%	ACT62322.1
HC2_GM001679	1,748,831:1750699	Triose-phosphate isomerase	Bacteria	99%	0	99.60%	WP_003643975.1
HC2_GM001680	1,750,743:1751351	Transcription elongation factor GreA	*L. plantarum*	99%	3.00E-100	100%	WP_063490463.1
HC2_GM001723	1,794,085:1794876	ABC transporter ATP-binding protein	*L. plantarum*	99%	0	99.24%	WP_063487840.1
HC2_GM001724	1,794,895:1796553	ABC transporter permease subunit	*L. plantarum*	99%	0	99.83%	WP_087615045.1
HC2_GM001760	1,835,087:1836274	Chaperonin GroEL	*L. plantarum*	99%	0	100%	WP_063852686.1
HC2_GM001785	1,864,019:1865083	Glucosamine-6-phosphate deaminase	*Lactobacillaceae*	99%	4.00E-173	100%	WP_003641817.1
HC2_GM001984	2,066,638:2068041	Transcription elongation factor GreA	*Lactobacillaceae*	99%	5.00E-97	99.38%	WP_003640314.1
HC2_GM002255	2,322,810:2323448	Orotate phosphoribosyltransferase	*Lactobacillaceae*	99%	4.00E-151	100%	WP_003642611.1
HC2_GM002331	2,399,389:2400078	Heat shock protein GrpE	*L. plantarum*	99%	3.00E-110	100%	ALV14711.1
HC2_GM002342	2,412,729:2415665	Cell surface protein	*L. plantarum*	99%	0	100%	AOB19372.1
HC2_GM002471	2,550,506:2551741	LPXTG cell wall anchor domain-containing protein	*Lactobacillaceae*	99%	0	100%	WP_003644794.1
HC2_GM002542	2,624,348:2624752	Membrane protein	*L. plantarum*	99%	1.00E-75	100%	WP_003643370.1
HC2_GM002619	2,710,817:2713045	BspA family leucine-rich repeat surface protein	*Lactobacillaceae*	99%	0	99.87%	WP_022638455.1
HC2_GM003060	3,160,616:3161197	Cell surface protein	*L. plantarum*	99%	4.00E-88	98.96%	WP_134902426.1
HC2_GM003061	3,161,278:3161628	LPXTG cell wall anchor domain-containing protein	*Lactobacillaceae*	99%	4.00E-71	100%	WP_003642858.1
HC2_GM003068	3,164,675:3165250	BspA family leucine-rich repeat surface protein	*L. plantarum*	99%	2.00E-136	100%	WP_003642866.1
HC2_GM003127	3,229,611:3231089	Cell surface protein	*Lactobacillaceae*	96%	0	100%	WP_033098925.1
HC2_GM003156	16,097:17626	Conjugal transfer protein	*L. plantarum*	99%	8.00E-70	100%	WP_180819087.1

HC2_GM003156 is located on plasmid 1; the other genes are located on the chromosome.

## 4. Discussion

Probiotics are widely used as a kind of alternatives to antibiotics (Parker, 1974; Zhang et al., 2018). Adhesion of probiotics to the gut mucus is the first step to exert different effects against the activity of pathogens (e.g., competition, exclusion, displacement, and repression) (Reid and Burton, 2002; Chen et al., 2007; Yadav et al., 2017). This study clarified the genes and proteins of *L. plantarum* HC-2 involved in adhesion to shrimp intestine and competitive exclusion against pathogenic *V. parahaemolyticus*. The results generally supported our core hypothesis that the shared *ftsH* gene could influence the adhesion of *L. plantarum* HC-2 to the shrimp mucus by regulating membrane proteins. Also, the genome of *L. plantarum* HC-2 harbors many genes relating to carbohydrate utilization and probiotic-host interactions.

Probiotic bacteria adhering to the gut may compete the colonized niches with pathogens, and thus prevent pathogen infestation (Servin, 2004; Fukuda, 2017). The major driving forces for enhancing bacterial adhesion are protein–protein and protein-carbohydrate interactions. The bacteria mainly share molecular mechanisms of initial adhesion to the host, and the presence of common adhesion proteins on the surface of bacteria could affect the adhesion-related molecules (Westerlund and Korhonen, 1993; Pizarro-Cerdá and Cossart, 2006; Kline et al., 2009; Iniguez-Palomares et al., 2011; Singh et al., 2018). *L. plantarum* HC-2 and *V. parahaemolyticus* can be adhered to the intestine of *Litopenaeus vannamei* (Sha et al., 2016a), thus the adhesion-related proteins encoded by homologous genes of these strains may compete for adhesion niches in the intestine as they may play a common functional role (Edgar and Batzoglou, 2006). In this study, homologous sequences obtained among *L. plantarum* HC-2 and all *V. parahaemolyticus* genomes were finally assigned to 11 genes which can attribute to the essential cellular metabolic processes. Several of them show multiple functions in different subcellular localization, like proteins related to transcription and translation which not only attribute to the essential cellular metabolic processes in cells but also act as adhesins when emerged on the cell surface of bacteria, such as genes numbered HC2_GM000845 (*rplF*), HC2_GM000995 (*prfC*), and HC2_GM000828 (*fusA*). Moreover, the gene numbered HC2_GM000464 was annotated as *ftsH* and was found that there was only one copy in this genome. The *ftsH* gene was firstly found in *Escherichia coli* and then proved in almost all bacteria to perform cellular roles for FtsH (Schumann, 1999). FtsH is a ubiquitous membrane-integrated protein, an ATP-and Zn^2+^-dependent metalloprotease, which belongs to ATPase associated with different cellular activities (AAA) protein family and in responsible for regulating the protein stability of specific and critical regulators to control cellular processes. Given the critical role of FtsH in cellular processes, the *ftsH* gene was selected as the target gene for mining adhesion-related biomarkers among 11 genes. In this study, the double-crossover mutagenesis was performed and obtained nearly 5,000 mutants one *ftsH* mutant. The number of mutants showed desired genotype only 0.02% due to the fact that the second homologous crossover is random. Thus, it is difficult to obtain the desired genotype at the absence of selection pressure, and a suitable method should be developed to get target mutagenesis of *L. plantarum*.

FtsH protease is very important for controlling the membrane protein, heat shock response, lipopolysaccharide biosynthesis, viral infection, and mediating colicin activity in *E. coli* (Schumann, 1999), and it could also regulate the cytoplasmic substrates in *Caulobacter crescentus* and *Bacillus subtilis* (Langklotz et al., 2012). However, the FtsH is dispensable for normal growth of bacteria except that survival upon stress conditions like heat shock or osmotic stress (Beier et al., 1997; Deuerling et al., 1997; Fischer et al., 2002; Lithgow et al., 2004; Fiocco et al., 2009; Langklotz et al., 2012). In *L. plantarum*, the FtsH mutant reduced the capacity to form biofilms and changed the physico-chemical surface properties, which possibly linked to the alterations of surface charge and/or architecture (Bove et al., 2012). Given that in present study, the bacteria were washed three times with phosphate buffer solution before being used to test their adhesion to mucus, and there was basically no difference in charge on the surface of cells. However, we found that the FtsH mutant increased the adhesion ability to the mucus (Garrett et al., 2008). This is consistent with previous studies, which identified that the down-expression of FtsH could promote biofilm formation in *L. plantarum* and *E. coli* (Langklotz et al., 2012; Gu et al., 2021). Therefore, the absence of *ftsH* in *L. plantarum* HC-2 might mainly alter the membrane architecture depending on the regulatory protease activity of FtsH (Garrett et al., 2008; Langklotz et al., 2012; Gu et al., 2021).

The obtained mutant strains increased the adhesion ability to mucus of shrimp, and comparative proteomic analysis was further performed to create a molecular map of the adhesion regulation processes. 1355 proteins were present in both groups, while 13 and 20 proteins were uniquely identified in wild type and mutant strains, respectively (Figure 1B). There was basically no change in the number of total proteins expressed, but the number of uniquely expressed proteins exceeded 1% in each group. A total of 80 differential expressed proteins were found, including 54 up-regulated proteins. Many increased functional activities in the mutant strains reveal that the improvement of *L. plantarum* HC-2 adhesion requires the mobilization of its more basic biological and cellular processes. In addition, the proteomic results showed that the expression of FtsH was still existed in the mutant strains with 10.56% down-regulation (data not shown) and showed no significant difference from the wild type strain, suggesting that the mutants were partially repaired. This speculation could be supported by the elevated abundance of proteins for reducing damage such as proteins involved in enzyme metabolism and DNA repair pathways (Figure 2). *L. acidophilus* also responded to oxidative stress in the similarly way (Calderini et al., 2017). However, further studies are needed to clarify how the missing *ftsH* gene is repaired. A 10.56% decrease in FtsH expression already altered the adhesion ability of this group, so the results in this study can be used for subsequent analysis to test our hypotheses.

Surface proteins play an important role in the adhesion of bacteria to mucus in two ways. The first one is that surface proteins are bound to the membrane by hydrophobic transmembrane domain(s) and lipoprotein, and the second is that surface proteins are attached to the cell surface by noncovalent or recognizing some cell-wall-binding domains (e.g., LPXTG, CWBD1, and GW proteins) (Deepika and Charalampopoulos, 2010). We found that the differentially expressed proteins were predicted to be located in the cytoplasm and membrane rather than the cell wall, suggesting that FtsH mainly affects the adhesion of *L. plantarum* by regulating the expression of the membrane proteins.

Cell division inhibitor MinD (protein ID: G0M1M7) participates in the division of cell, and the overexpression of MinD could result in filamentation of cell by enhancing MinC-mediated inhibition of cell division (Nguyen et al., 2013). However, the *minC* gene was identified as antimicrobial activities other than being involved in cell division in *L. acidophilus* (Nguyen, 2012; Nguyen et al., 2013). Thus, the members of cell division inhibitors may play different roles in different strains. In this study, the increased MinD in membrane may result in filamentation of cell which may participate in the adhesion as an anchoring site for other adhesion molecules. The protein I9L3P8 encoded by *choS* was reported to have a compatible solutes glycine betaine/carnitine/choline transport system (Rossi et al., 2016). Moreover, choline was found to be a direct precursor of phosphatidylcholine, which was a major lipid membrane component in the Rhizobiaceae family (Dupont et al., 2004). The *choS* was also involved in tolerance to stress from the external environment (Rossi et al., 2016), so the higher expression of gene encoding for I9L3P8 may be a response to protect itself against pathogens and enhance adhesion to the host. The A0A2S9W1Y1 is annotated as ATP synthase subunit a, which is a membrane-bound stator subunit of the ATP synthase and is essential for proton translocation (Ishmukhametov et al., 2017). These three proteins were significantly up-regulated in *L. plantarum* HC-2 when it cocultured with *V. parahaemolyticus* E1 for 6 h and 12 h while down-regulated at other time points. Biological membranes are a mix of membrane proteins and their lipid partners. One potential consequence of membrane crowding is that this conformational change could cause a change in free energy that is associated with the area available to the rest of the proteins, resulting in depletion forces (Phillips et al., 2009). These changes play a critical role in sensory and signaling pathways. Therefore, during this experiment, it was observed that the expression of genes can at times be up or down. The similar gene-expression patterns were also found in *Pseudomonas.syringae* pv. *actinidiae* during the first five days after infection of kiwifruit (McAtee et al., 2018), in which the expression of several genes from the plasmid-borne putative aromatic pathway showed a fluctuating pattern over the infection time course. In addition, compared with *V. parahaemolyticus* E1 after incubation alone, *aphA* in *V. parahaemolyticus* E1 was also observed to be up-regulated at 2 h and 6 h but down-regulated at 4 h and 8 h when *V. parahaemolyticus* E1 was cocultured with the same number of its own cells at 28°C (Sha et al., 2016a). The probiotics and pathogens share similar nutritional substrates, especially the amino acids. Thus, the gene encoding amino acid permeases (A0A241RSP9) was induced at earlier time. As above mentioned, four proteins (G0M1M7, I9L3P8, A0A2S9W1Y1, A0A241RSP9) may be involved in the competitive exclusion against pathogens in the mucus of shrimp by regulating their quantity of proteins on the surface of *L. plantarum* HC-2.

The application of strains is benefit from their adaption to the host intestinal environment and interaction with the host. As a kind of facultatively heterofermentative LAB, *L. plantarum* HC-2 genome possesses genes for both the phosphoketolase and Embden-Meyerhof pathways (EMP). Thus, it mainly ferments carbohydrate *via* EMP by using glucose. However, in the absence of six-carbon sugars, it can also ferment five-carbon carbohydrates such as arabinose or ribose *via* the phosphoketolase pathway (Bustos et al., 2005; Abriouel et al., 2017). As animals have limited evolved abilities to digest complex polysaccharides (Cantarel et al., 2012), the enzymes produced by intestinal microorganisms are needed to metabolize carbohydrate (e.g., xylan, β-glucan, cellulose, and chitosan). The genes encoding carbohydrate-modifying enzymes could be used to predict the possible adaption and enrichment of bacteria in the intestine (Abriouel et al., 2017). In present study, many enzymes involved in degrading of xylan and β-glucan are found in *L. plantarum* HC-2 genome. As the accessory enzymes for the degradation of xylan, acetyl xylan esterase (four genes, Table 3), glycerol-3-phosphate cytidylyltransferase, eukaryotic-like serine/threonine-protein kinase, xylan endo-1,3-beta-xylosidase activity and xylanase were predicted in *L. plantarum* HC-2 genome sequence, which can remove the terminal acetate residues from xylan polymers and act in the xylan catabolic pathway (Kim et al., 2020). Furthermore, 12 genes coding for enzymes involved in the degradation of beta-glucan were also predicted in *L. plantarum* HC-2 (Supplementary Table S3). Xylan occurs in nearly all plants and some marine algae, and could act as immune modulators to enhance animal immunity (Yang et al., 2019). However, as a kind of soluble non-starch polysaccharides (SNPs), the presence of xylan in diet could also interfere with feed utilization and negatively affect animal performance (Sinha et al., 2011; Yao et al., 2019). Beta-glucan often used as a feed additive in aquaculture, which can enhance fish and shrimp innate immune responses (Chang et al., 2003; Yamamoto et al., 2018), but it is also a kind of SNPs. Therefore, the presence of these enzymes is predicted to contribute to the degradation of xylan and beta-glucan in the intestine. Interestingly, the *L. plantarum* HC-2 genome has 22 genes responsible for the cellulose synthesis, including cellulose synthase (Supplementary Table S3). The cellulose synthase (17 genes) could accumulate cellulose on the cell wall surface as an extracellular matrix for adhesion of cell and formation of biofilm for protecting the bacteria (Abriouel et al., 2017). Over 7% (229 of 3,259 genes) of the identified genes in *L. plantarum* HC-2 are involved in carbohydrate metabolism, which is similar to the genome of *L. pentosus* MP-10 (Abriouel et al., 2017). The abundance of carbohydrate metabolism genes is important for *L. plantarum* HC-2 to adapt the intestinal environment, which may enhance its possibilities of survival and persistence and competitiveness to pathogens within the intestine of host.

Probiotic lactobacilli can express adhesion-related proteins to mimic the colonization process of pathogens, resulting in several probiotic effects. The identification and characterization of these proteins, often strain-specific, involved in interaction with the host are necessary to evaluate *a priori* the probiotic potential of *Lactobacillus* sp. Candidates (Abriouel et al., 2017). In this study, the possible interaction of *L. plantarum* HC-2 with the host was bioinformatically predicted from the genome sequence. Several adhesion-related proteins were predicted, such as cell surface proteins, LPXTG cell wall anchor domain-containing proteins and elongation factors. However, the role of these proteins in *L. plantarum* HC-2 has not yet been determined, requiring further mutation or proteomic studies for this purpose.

In summary, this study identified and clarified the underlying mechanisms of genes and proteins of *L. plantarum* HC-2 involved in adhesion to intestine and competitive exclusion against pathogenic *V. parahaemolyticus*. The findings advance our mechanistic understanding of the selective adhesion of probiotics and competitive exclusion of pathogens. Also, the repertoire of genes identified in the *L. plantarum* HC-2 genome have an important implications for its applications in other animals as a potential probiotic.

### 4.1. Renamed strain HC-2 statement

The strain HC-2 was previously identified as *Lactobacillus pentosus* (now NCBI changed the name of *Lactobacillus pentosus* to *Lactiplantibacillus pentosus*) based on the analysis of partial 16S rRNA sequence which was more similar to *L. pentosus* JCM 1558, and therefore named as *L. pentosus* HC-2. However, now the complete genome of strain HC-2 was obtained, and the phylogenetic tree was constructed based on the genomes of strain HC-2, 5 strains from *L. plantarum* and 5 strains from *L. pentosus* (Supplementary Figure S2). The result showed that HC-2 clustered well with *L. plantarum* strains, so the strain HC-2 was renamed as *L. plantarum* HC-2 from now on. This result was also confirmed by calculating ANI (Average Nucleotide Identity)^3^ of strain HC-2 with *L. plantarum* or *L. pentosus* and estimating DNA–DNA relatedness using the Genome-to-Genome Distance Calculator (GGDC) 3.0 provided by the DSMZ^4^ (Supplementary Table S4).

## Data availability statement

The datasets presented in this study can be found in online repositories. The names of the repository/repositories and accession number(s) can be found at: https://www.ncbi.nlm.nih.gov/, SRR21010423. http://www.proteomexchange.org/, PXD036035. https://www.ncbi.nlm.nih.gov/, PRJNA868485.

## Ethics statement

The animal study was reviewed and approved by Science and Technology Ethics Committee of Dezhou University (Dezhou University, Dezhou, China).

## Author contributions

YS: conceptualization, data curation, formal analysis, funding acquisition, investigation, methodology, resources, supervision, visualization, writing—original draft, and writing—review and editing. QY: formal analysis, writing—original draft, and visualization. JL: methodology and funding acquisition. JY: conceptualization and writing—review and editing. SX: writing—review and editing. ZH: writing—review and editing. JR: conceptualization and writing—review and editing. JQ: conceptualization and writing—review and editing. SZ: writing—review and editing. GW: data curation and methodology. WD: data curation and methodology. All authors contributed to the article and approved the submitted version.

## Funding

This work was supported by the National Natural Science Foundation of China (31802309), the Natural Science Foundation of Shandong Province (CN) (ZR2017BC027), Research Foundation for Advanced Talents of Dezhou University (2019xgrc35).

## Conflict of interest

The authors declare that the research was conducted in the absence of any commercial or financial relationships that could be construed as a potential conflict of interest.

## Publisher’s note

All claims expressed in this article are solely those of the authors and do not necessarily represent those of their affiliated organizations, or those of the publisher, the editors and the reviewers. Any product that may be evaluated in this article, or claim that may be made by its manufacturer, is not guaranteed or endorsed by the publisher.

## Footnotes

^1^http://www.uniprot.org/

^2^http://string.embl.de/

^3^www.ezbiocloud.net/tools/ani

^4^http://ggdc.dsmz.de/distcalc2.php

## References

[ref1] Aaron C. E. Darling B. M. Blattner F. R. Perna N. T. (2004). Mauve: multiple alignment of conserved genomic sequence with rearrangements. Genome Res. 14, 1394–1403. doi: 10.1101/, PMID: 15231754 10.1101/gr.2289704 PMC442156

[ref2] Abriouel H. Perez Montoro B. Casimiro-Soriguer C. S. Perez Pulido A. J. Knapp C. W. Caballero Gomez N.. (2017). Insight into potential probiotic markers predicted in *Lactobacillus pentosus* MP-10 genome sequence. Front. Microbiol. 8:891. doi: 10.3389/fmicb.2017.00891, PMID: 28588563 PMC5439011

[ref3] Beier D. Spohn G. Rappuoli R. Scarlato V. (1997). Identification and characterization of an operon of *Helicobacter pylori* that is involved in motility and stress adaptation. J. Bacteriol. 179, 4676–4683. doi: 10.1128/jb.179.15.4676-4683.1997, PMID: 9244252 PMC179311

[ref4] Bove P. Capozzi V. Garofalo C. Rieu A. Spano G. Fiocco D. (2012). Inactivation of the ftsH gene of *Lactobacillus plantarum* wcfs1: effects on growth, stress tolerance, cell surface properties and biofilm formation. Microbiol. Res. 167, 187–193. doi: 10.1016/j.micres.2011.07.001 21795030

[ref5] Bradford M. M. (1976). A rapid and sensitive method for the quantitation of microgram quantities of protein utilizing the principle of protein-dye binding. Anal. Biochem. 72, 248–254. doi: 10.1016/0003-2697(76)90527-3, PMID: 942051

[ref6] Bustos G. Moldes A. B. Cruz J. M. Domínguez J. M. (2005). Influence of the metabolism pathway on lactic acid production from hemicellulosic trimming vine shoots hydrolyzates using *Lactobacillus pentosus*. Biotechnol. Prog. 21, 793–798. doi: 10.1021/bp049603v, PMID: 15932258

[ref7] Calderini E. Celebioglu H. U. Villarroel J. Jacobsen S. Svensson B. Pessione E. (2017). Comparative proteomics of oxidative stress response of *Lactobacillus acidophilus* NCFM reveals effects on DNA repair and cysteine *de novo* synthesis. Proteomics 17:178. doi: 10.1002/pmic.201600178 28045221

[ref8] Cantarel B. L. Lombard V. Henrissat B. (2012). Complex carbohydrate utilization by the healthy human microbiome. PLoS One 7:e28742. doi: 10.1371/journal.pone.0028742, PMID: 22719820 PMC3374616

[ref9] Chang C.-F. Su M.-S. Chen H.-Y. Liao I.-C. (2003). Dietary β-1, 3-glucan effectively improves immunity and survival of *Penaeus monodon* challenged with white spot syndrome virus. Fish Shellfish Immunol. 15, 297–310. doi: 10.1016/S1050-4648(02)00167-5, PMID: 12969651

[ref10] Chen X. Xu J. Shuai J. Chen J. Zhang Z. Fang W. (2007). The s-layer proteins of *Lactobacillus crispatus* strain zj001 is responsible for competitive exclusion against *Escherichia coli* o157: H7 and *Salmonella typhimurium*. Int. J. Food Microbiol. 115, 307–312. doi: 10.1016/j.ijfoodmicro.2006.11.007, PMID: 17289201

[ref11] De Angelis M. Calasso M. Cavallo N. Di Cagno R. Gobbetti M. (2016). Functional proteomics within the genus Lactobacillus. Proteomics 16, 946–962. doi: 10.1002/pmic.201500117 27001126

[ref12] Deepika G. Charalampopoulos D. (2010). Surface and adhesion properties of lactobacilli. Adv. Appl. Microbiol. 70, 127–152. doi: 10.1016/s0065-2164(10)70004-6 20359456

[ref13] Deuerling E. Mogk A. Richter C. Purucker M. Schumann W. (1997). The ftsH gene of *Bacillus subtilis* is involved in major cellular processes such as sporulation, stress adaptation and secretion. Mol. Microbiol. 23, 921–933. doi: 10.1046/j.1365-2958.1997.2721636.x, PMID: 9076729

[ref14] Du Y. Zhou S. Liu M. Wang B. Jiang K. Fang H.. (2019). Understanding the roles of surface proteins in regulation of *Lactobacillus pentosus* HC-2 to immune response and bacterial diversity in midgut of *Litopenaeus vannamei*. Fish Shellfish Immunol. 86, 1194–1206. doi: 10.1016/j.fsi.2018.12.073, PMID: 30599258

[ref15] Dupont L. Garcia I. Poggi M. C. Alloing G. Mandon K. Le Rudulier D. (2004). The *Sinorhizobium meliloti* abc transporter cho is highly specific for choline and expressed in bacteroids from *Medicago sativa* nodules. J. Bacteriol. 186, 5988–5996. doi: 10.1128/JB.186.18.5988-5996.2004, PMID: 15342567 PMC515146

[ref16] Edgar R. C. Batzoglou S. (2006). Multiple sequence alignment. Curr. Opin. Struct. Biol. 16, 368–373. doi: 10.1016/j.sbi.2006.04.004 16679011

[ref17] Fang H. Wang B. Jiang K. Liu M. Wang L. (2020). Effects of *Lactobacillus pentosus* HC-2 on the growth performance, intestinal morphology, immune-related genes and intestinal microbiota of *Penaeus vannamei* affected by aflatoxin B1. Aquaculture 525:735289. doi: 10.1016/j.aquaculture.2020.735289

[ref18] Fiocco D. Collins M. Muscariello L. Hols P. Kleerebezem M. Msadek T.. (2009). The *Lactobacillus plantarum* ftsH gene is a novel member of the CtsR stress response regulon. J. Bacteriol. 191, 1688–1694. doi: 10.1128/JB.01551-08, PMID: 19074391 PMC2648225

[ref19] Fischer B. Rummel G. Aldridge P. Jenal U. (2002). The FtsH protease is involved in development, stress response and heat shock control in *Caulobacter crescentus*. Mol. Microbiol. 44, 461–478. doi: 10.1046/j.1365-2958.2002.02887.x 11972783

[ref20] Fukuda K. (2017). Is it feasible to control pathogen infection by competitive binding of probiotics to the host? Virulence 8, 1502–1505. doi: 10.1080/21505594.2017.1382798, PMID: 28934003 PMC5810465

[ref21] Garrett T. R. Bhakoo M. Zhang Z. (2008). Bacterial adhesion and biofilms on surfaces. Prog. Nat. Sci. 18, 1049–1056. doi: 10.1016/j.pnsc.2008.04.001

[ref22] Gu Y. Tian J. Zhang Y. Wu R. Li L. Zhang B.. (2021). Dissecting signal molecule AI-2 mediated biofilm formation and environmental tolerance in *Lactobacillus plantarum*. J. Biosci. Bioeng. 131, 153–160. doi: 10.1016/j.jbiosc.2020.09.015, PMID: 33077360

[ref23] Iniguez-Palomares C. Jimenez-Flores R. Vazquez-Moreno L. Ramos-Clamont-Montfort G. Acedo-Felix E. (2011). Protein-carbohydrate interactions between Lactobacillus salivarius and pig mucins. J. Anim. Sci. 89, 3125–3131. doi: 10.2527/jas.2010-2996 21622872

[ref24] Ishmukhametov R. R. DeLeon-Rangel J. Zhu S. Vik B. S. (2017). Analysis of an N-terminal deletion in subunit a of the *Escherichia coli* ATP synthase. J. Bioenerg. Biomembr. 49, 171–181. doi: 10.1007/s10863-017-9694-z, PMID: 28078625 PMC5376380

[ref25] Kankainen M. Paulin L. Tynkkynen S. von Ossowski I. Reunanen J. Partanen P.. (2009). Comparative genomic analysis of *Lactobacillus rhamnosus* GG reveals pili containing a human-mucus binding protein. P. Natl. A. Sci. 106, 17193–17198. doi: 10.1073/pnas.0908876106, PMID: 19805152 PMC2746127

[ref27] Kim M.-J. Jang M.-U. Nam G.-H. Shin H. Song J.-R. Kim T.-J. (2020). Functional expression and characterization of acetyl xylan esterases ce family 7 from *Lactobacillus antri* and *Bacillus halodurans*. J. Microbiol. Biotechnol. 30, 155–162. doi: 10.4014/jmb.2001.01004, PMID: 31986559 PMC9728288

[ref28] Kline K. A. Fälker S. Dahlberg S. Normark S. Henriques-Normark B. (2009). Bacterial adhesins in host-microbe interactions. Cell Host Microbe 5, 580–592. doi: 10.1016/j.chom.2009.05.011 19527885

[ref29] Langklotz S. Baumann U. Narberhaus F. (2012). Structure and function of the bacterial AAA protease FtsH. Biochim. Biophys. Acta 1823, 40–48. doi: 10.1016/j.bbamcr.2011.08.015, PMID: 21925212

[ref30] Lithgow J. K. Ingham E. Foster S. J. (2004). Role of the hprT–ftsH locus in *Staphylococcus aureus*. Microbiology 150, 373–381. doi: 10.1099/mic.0.26674-0 14766915

[ref31] McAtee P. Brian L. Curran B. Linden O. Nieuwenhuizen J. Chen X. (2018). Re-programming of *Pseudomonas syringae* pv. Actinidiae gene expression during early stages of infection of kiwifruit. BMC Genomics 19:822. doi: 10.1186/s12864-018-5197-5, PMID: 30442113 PMC6238374

[ref32] Meng J. Wang Y.-Y. Hao Y.-P. (2021). Protective function of surface layer protein from *Lactobacillus casei* fb05 against intestinal pathogens *in vitro*. Biochem. Biophys. Res. Commun. 546, 15–20. doi: 10.1016/j.bbrc.2021.01.101 33561743

[ref33] Minj J. Chandra P. Paul C. Sharma R. K. (2021). Bio-functional properties of probiotic Lactobacillus: current applications and research perspectives. Crit. Rev. Food Sci. Nutr. 61, 2207–2224. doi: 10.1080/10408398.2020.1774496, PMID: 32519883

[ref34] Nguyen T. (2012). Cell division inhibitor minC from *Lactobacillus acidophilus* VTCC-B-871 and detection of antimicrobial activities in functional study. Afr. J. Pharm. Pharmaco. 6, 3293–3298. doi: 10.5897/ajpp12.1476

[ref35] Nguyen T. H. Doan V. T. Ha L. D. Nguyen H. N. (2013). Molecular cloning, expression of mind gene from *Lactobacillus acidophilus* VTCC-B-871 and analyses to identify *Lactobacillus rhamnosus* pn04 from Vietnam hottuynia cordata thunb. Indian J. Microbiol. 53, 385–390. doi: 10.1007/s12088-013-0384-1, PMID: 24426140 PMC3779299

[ref36] Okano K. Zhang Q. Shinkawa S. Yoshida S. Tanaka T. Fukuda H.. (2009). Efficient production of optically pure D-lactic acid from raw corn starch by using a genetically modified L-lactate dehydrogenase gene-deficient and α-amylase-secreting *Lactobacillus plantarum* strain. Appl. Environ. Microbiol. 75, 462–467. doi: 10.1128/AEM.01514-08, PMID: 19011066 PMC2620712

[ref37] O'sullivan O. O'Callaghan J. Sangrador-Vegas A. McAuliffe O. Slattery L. Kaleta P.. (2009). Comparative genomics of lactic acid bacteria reveals a niche-specific gene set. BMC Microbiol. 9, 1–9. doi: 10.1186/1471-2180-9-50 19265535 PMC2660350

[ref38] Parker R. (1974). Probiotics, the other half of the antibiotic story. Anim. Nutr. Health 29, 4–8.

[ref39] Partrick K. A. Rosenhauer A. M. Auger J. Arnold A. R. Ronczkowski N. M. Jackson L. M.. (2021). Ingestion of probiotic (*Lactobacillus helveticus* and *Bifidobacterium longum*) alters intestinal microbial structure and behavioral expression following social defeat stress. Sci. Rep. 11, 1–12. doi: 10.1038/s41598-021-83284-z 33580118 PMC7881201

[ref40] Phillips R. Ursell T. Wiggins P. Sens P. (2009). Emerging roles for lipids in shaping membrane-protein function. Nature 459, 379–385. doi: 10.1038/nature08147, PMID: 19458714 PMC3169427

[ref41] Pizarro-Cerdá J. Cossart P. (2006). Bacterial adhesion and entry into host cells. Cells 124, 715–727. doi: 10.1016/j.cell.2006.02.012 16497583

[ref42] Reid G. Burton J. (2002). Use of Lactobacillus to prevent infection by pathogenic bacteria. Microb. Infect. 4, 319–324. doi: 10.1016/S1286-4579(02)01544-7 11909742

[ref43] Rossi F. Zotta T. Iacumin L. Reale A. (2016). Theoretical insight into the heat shock response (HSR) regulation in Lactobacillus casei and *L. rhamnosus*. J. Theor. Biol. 402, 21–37. doi: 10.1016/j.jtbi.2016.04.029 27142777

[ref44] Sanders M. E. Benson A. Lebeer S. Merenstein D. J. Klaenhammer T. R. (2018). Shared mechanisms among probiotic taxa: implications for general probiotic claims. Curr. Opin. Biotechnol. 49, 207–216. doi: 10.1016/j.copbio.2017.09.007, PMID: 29128720

[ref45] Schumann W. (1999). FtsH – a single-chain charonin? FEMS Microbiol. Rev. 23, 1–11. doi: 10.1016/S0168-6445(98)00024-2, PMID: 10077851

[ref46] Servin A. L. (2004). Antagonistic activities of lactobacilli and Bifidobacteria against microbial pathogens. FEMS Microbiol. Rev. 28, 405–440. doi: 10.1016/j.femsre.2004.01.003, PMID: 15374659

[ref47] Sha Y. (2016) Studies on probiotic mechannism of lactic acid bacteria in *Litopenaeus vannamei*. [dissertation/doctoral thesis]. Institute of Oceanology, Chinese Acadamy of sciences.

[ref48] Sha Y. Wang B. Liu M. Jiang K. Wang L. (2016a). Interaction between *Lactobacillus pentosus* HC-2 and *Vibrio parahaemolyticus* E1 in *Litopenaeus vannamei* in vivo and in vitro. Aquaculture 465, 117–123. doi: 10.1016/j.aquaculture.2016.09.007

[ref49] Sha Y. Wang L. Liu M. Jiang K. Xin F. Wang B. (2016b). Effects of lactic acid bacteria and the corresponding supernatant on the survival, growth performance, immune response and disease resistance of *Litopenaeus vannamei*. Aquaculture 452, 28–36. doi: 10.1016/j.aquaculture.2015.10.014

[ref50] Singh K. S. Kumar S. Mohanty A. K. Grover S. Kaushik J. K. (2018). Mechanistic insights into the host-microbe interaction and pathogen exclusion mediated by the mucus-binding protein of *Lactobacillus plantarum*. Sci. Rep. 8:14198. doi: 10.1038/s41598-018-32417-y, PMID: 30242281 PMC6155027

[ref51] Sinha A. K. Kumar V. Makkar H. P. De Boeck G. Becker K. (2011). Non-starch polysaccharides and their role in fish nutrition–a review. Food Chem. 127, 1409–1426. doi: 10.1016/j.foodchem.2011.02.042

[ref52] Teame T. Wang A. Xie M. Zhang Z. Yang Y. Ding Q.. (2020). Paraprobiotics and postbiotics of probiotic lactobacilli, their positive effects on the host and action mechanisms: a review. Front. Nutr. 7:570344. doi: 10.3389/fnut.2020.570344, PMID: 33195367 PMC7642493

[ref53] Van den Abbeele P. Marzorati M. Derde M. De Weirdt R. Joan V. Possemiers S.. (2016). Arabinoxylans, inulin and Lactobacillus reuteri 1063 repress the adherent-invasive *Escherichia coli* from mucus in a mucosa-comprising gut model. NPJ Biofilms Microbi. 2:16016. doi: 10.1038/npjbiofilms.2016.16 PMC5515265 28721250

[ref54] Van Tassell M. L. Miller M. J. (2011). Lactobacillus adhesion to mucus. Nutrients 3, 613–636. doi: 10.3390/nu3050613, PMID: 22254114 PMC3257693

[ref55] Wang H. Niu Y. Pan J. Li Q. Lu R. (2020). Antibacterial effects of *Lactobacillus acidophilus* surface-layer protein in combination with nisin against *Staphylococcus aureus*. LWT 124:109208. doi: 10.1016/j.lwt.2020.109208

[ref56] Wang J. Zhang J. Liu W. Zhang H. Sun Z. (2021). Metagenomic and metatranscriptomic profiling of *Lactobacillus casei* Zhang in the human gut. NPJ Biofilms Microbi. 7:55. doi: 10.1038/s41522-021-00227-2, PMID: 34210980 PMC8249650

[ref57] Westerlund B. Korhonen T. (1993). Bacterial proteins binding to the mammalian extracellular matrix. Mol. Microbiol. 9, 687–694. doi: 10.1111/j.1365-2958.1993.tb01729.x, PMID: 7901732

[ref58] Yadav A. K. Tyagi A. Kumar A. Panwar S. Grover S. Saklani A. C.. (2017). Adhesion of lactobacilli and their anti-infectivity potential. Crit. Rev. Food Sci. Nutr. 57, 2042–2056. doi: 10.1080/10408398.2014.918533, PMID: 25879917

[ref59] Yamamoto F. Y. Yin F. Rossi W. Jr. Hume M. Gatlin D. M. III (2018). β-1, 3 glucan derived from Euglena gracilis and Algamune™ enhances innate immune responses of red drum (*Sciaenops ocellatus* L.). Fish Shellfish Immunol. 77, 273–279. doi: 10.1016/j.fsi.2018.04.003, PMID: 29625243

[ref60] Yang P. Hu H. Li Y. Ai Q. Zhang W. Zhang Y.. (2019). Effect of dietary xylan on immune response, tight junction protein expression and bacterial community in the intestine of juvenile turbot (*Scophthalmus maximus* L.). Aquaculture 512:734361. doi: 10.1016/j.aquaculture.2019.734361

[ref61] Yao W. Li X. Chowdhury M. K. Wang J. Leng X. (2019). Dietary protease, carbohydrase and micro-encapsulated organic acid salts individually or in combination improved growth, feed utilization and intestinal histology of pacific white shrimp. Aquaculture 503, 88–95. doi: 10.1016/j.aquaculture.2018.12.064

[ref62] Zhang H. Liu T. Zhang Z. Payne S. H. Zhang B. McDermott J. E.. (2016). Integrated proteogenomic characterization of human high-grade serous ovarian cancer. Cells 166, 755–765. doi: 10.1016/j.cell.2016.05.069 PMC4967013 27372738

[ref63] Zhang Z. Lv J. Pan L. Zhang Y. (2018). Roles and applications of probiotic Lactobacillus strains. Appl. Microbiol. Biotechnol. 102, 8135–8143. doi: 10.1007/s00253-018-9217-9, PMID: 30032432

