# Supplementary material for: Homologous genes shared between probiotics and pathogens affect the adhesion of probiotics and exclusion of pathogens in the gut mucus of shrimp
Source: Front Microbiol. 2023 Jun 14;14:1195137. doi: 10.3389/fmicb.2023.1195137 (PMC10301755; doi:10.3389/fmicb.2023.1195137)
Supplement: Supplementary file 1 [file Data_Sheet_1.docx]

***Supplementary Material***

**Homologous genes shared between probiotics and pathogens affect the adhesion of probiotics and exclusion of pathogens in the gut mucus of shrimp**

**1 Supplementary Tables**

Table S1 The genomes used in this study.

| Strain | GenBank Number | Strain | GenBank Number |
| --- | --- | --- | --- |
| The identifier of *Vibrio parahaemolyticus* for the genome assembly | | | |
| MAVP-Q  (SAMN05579852) | GCA_002209725.2 | FORC_006 | GCA_001304775.1 |
| BB22OP | GCA_000328405.1 | FORC_004 | GCA_001433415.1 |
| MAVP-R | GCA_002220985.3 | FDAARGOS_51 | GCA_001188185.2 |
| S107-1 | GCA_003047085.1 | FORC_008 | GCA_001244315.1 |
| R14 | GCA_003076895.1 | PB1937 | GCA_003351885.1 |
| 20130629002S01 | GCA_003085735.1 | FORC_072 | GCA_003612695.1 |
| R13 | GCA_003119375.1 | FORC_018 | GCA_001887055.1 |
| RIMD 2210633 substr. RIMD 2210633 | GCA_000196095.1 | FORC_071 | GCA_003612715.1 |
| ATCC 17802 | GCA_001558495.2 | FDA_R31 | GCA_000430405.1 |
| FORC_014 | GCA_001636035.1 | 160807 | GCA_003691525.1 |
| MAVP-Q  (SAMN03766034) | GCA_001682175.1 | VPD14 | GCA_004006515.1 |
| CHN25 | GCA_001700835.1 | D3112 | GCA_004194515.1 |
| FORC_023 | GCA_001758605.1 | CDC_K4557 | GCA_000430425.1 |
| FORC_022 | GCA_001879585.1 | UCM-V493 | GCA_000568495.1 |
| FDAARGOS_191 | GCA_002073775.2 |  |  |
| The identifier of *Lactiplantibacillus pentosus* for the genome assembly | | | |
| DSM20314 | [GCA_003641185.1](https://www.ncbi.nlm.nih.gov/assembly/GCA_003641185.1) | ZFM94 | [GCA_003627375.1](https://www.ncbi.nlm.nih.gov/assembly/GCA_003627375.1) |
| BGM48 | [GCA_002850015.1](https://www.ncbi.nlm.nih.gov/assembly/GCA_002850015.1) | ZFM222 | [GCA_003627295.1](https://www.ncbi.nlm.nih.gov/assembly/GCA_003627295.1) |
| SLC13 | [GCA_002211885.1](https://www.ncbi.nlm.nih.gov/assembly/GCA_002211885.1) |  |  |
| The identifier of *Lactiplantibacillus plantarum* for the genome assembly | | | |
| DMC-S1 | GCA_029855105.1 | BK-021 | GCA_013487805.1 |
| PC520 | GCA_002576835.1 | L55 | GCA_026153115.1 |
| ATCC 202195 | GCA_018351295.1 |  |  |

Table S2 Primers used in this study.

| Protein IDs or genes | Sequences of primers (5’-3’) |
| --- | --- |
| *ftsH*up-F-XhoI | GCTGCTCGAGGGGGTAAAAAGTTAATGCCC |
| *ftsH*up-R | CCCAGCTCCTTCATTTTACGCACATTTGACTATCATTGACC |
| *ftsH*do-F | GGTCAATGATAGTCAAATGTGCGTAAAATGAAGGAGCTGGG |
| *ftsH*do-R-EcoRI | CTCGGAATTCTAGTTCGCGTTCACTGAACTG |
| Test*ftsH*-F2 | GGAGTACATCTGAAACGATG |
| Test*ftsH*-R2 | CCTTCACATCGATCTTATGC |
| I9L3P8-F | CTGAATTTACTGGCTCGGTGCTGGA |
| I9L3P8-R | GCTGATTGTCTTCAAATGATGTTCT |
| I8R6Y9-F | GGTGCTTGGGAGAAATTACCTGGTC |
| I8R6Y9-R | GCGGCACTGTCATACTTCCCATTGT |
| F6ISB0-F | ATTGCCCATCATAGTCGGTT |
| F6ISB0-R | GTCATCCCCATCCCCTTAC |
| A0A241RSP9-F | GCTTTGTGGCTGGATGGACG |
| A0A241RSP9-R | TTCACCGAACGCTGAGACATT |
| F6IZF7-F | AGTGACGGTGATTTTGCTACTCCTT |
| F6IZF7-R | CGCAATCAGCATCAGTCCAAT |
| G0M1M7-F | CACAAAATGCGGACAAGGATG |
| G0M1M7-R | GTTCCAATAAGCCAACGACCC |
| I9AKC3-F | CTGAGTGTCGTGGTGGTTGGT |
| I9AKC3-R | TCGATGGCATCTTGTTCCTTG |
| A0A2S9W1Y1-F | CGTATCTGAAGAGTCCAACCGCCGA |
| A0A2S9W1Y1-R | TCCCGAACAGCCGAAGACCTAAAGT |
| *MA1*-F | CTCAAAACTAAACAAAGTTTC |
| *MA1*-R | CTTGTACACACCGCCCGTCA |
| *pvuA*-F | CAAACTCACTCAGACTCCA |
| *pvuA*-R | CGAACCGATTCAACACG |

Table S3 Putative carbohydrate-modifying enzymes identified in the genome of *Lactiplantibacillus plantarum* HC-2.

| Carbohydrate | Enzyme | Gene | Gene ID | Locus | EC number | CAZy Family |
| --- | --- | --- | --- | --- | --- | --- |
| xylan | acetyl xylan esterase |  | HC2_GM000172 | 191594:192586 | 3.1.1.72 | CE12 |
|  | glycerol-3-phosphate cytidylyltransferase | *tagD* | HC2_GM000990 | 1044039:1044437 | 2.7.7.39 | NA |
|  | eukaryotic-like serine/threonine-protein kinase | *stkP* | HC2_GM001342 | 1404110:1406134 | 2.7.11.1 | CBM2 |
|  | acetyl xylan esterase |  | HC2_GM002010 | 2093643:2094509 | 3.1.1.72 | CE1, CE4, CE12 |
|  | acetyl xylan esterase |  | HC2_GM002971 | 3060376:3061398 | 3.1.1.72 | CE1, CE4, CE12 |
|  | acetyl xylan esterase |  | HC2_GM002986 | 3075999:3076790 | 3.1.1.72 | CE1, CE4, CE12 |
|  | xylan endo-1,3-beta-xylosidase activity |  | HC2_GM003171 | Plas1:28905:29411 |  |  |
|  | xylanase |  | HC2_GM003235 | Plas2:45849:46964 | 3.2.1.8 | GH8 |
| cellulose | UDP-glucose 4-epimerase | *galE* | HC2_GM002963 | 3047860:3048864 | 5.1.3.2 | GT2 |
|  | Cellulose biosynthesis protein BcsQ |  | HC2_GM003175 | Plas1:31686:32486 |  |  |
|  | Cellulose biosynthesis protein BcsQ |  | HC2_GM003217 | Plas2:23585:24370 |  |  |
|  | cellulose synthase |  | HC2_GM000352 | 368002:369201 | 2.4.1.12 | GT2 |
|  | UDP-glucose 4-epimeras | *galE* | HC2_GM000545 | 577581:578576 | 5.1.3.2 | GT2 |
|  | cellulose synthase |  | HC2_GM000594 | 627951:629177 | 2.4.1.12 | GT2 |
|  | cellulose synthase |  | HC2_GM000595 | 629200:631260 | 2.4.1.12 | GT2 |
|  | cellulose synthase |  | HC2_GM000958 | 1011434:1012330 | 2.4.1.12 | GT2 |
|  | cellulose synthase |  | HC2_GM000959 | 1012348:1013412 | 2.4.1.12 | GT2 |
|  | cellulose synthase |  | HC2_GM000962 | 1015632:1016525 | 2.4.1.12 | GT2 |
|  | cellobiohydrolase |  | HC2_GM001009 | 1066049:1068268 | 3.2.1.4 | GH6 |
|  | cellulose synthase |  | HC2_GM001144 | 1221657:1222601 | 2.4.1.12 | GT2 |
|  | cellulose synthase |  | HC2_GM001146 | 1224571:1225599 | 2.4.1.12 | GT2 |
|  | cellulose synthase |  | HC2_GM001261 | 1326272:1327576 | 2.4.1.12 | GT2 |
|  | eukaryotic-like serine/threonine-protein kinase | *stkP* | HC2_GM001342 | 1404110:1406134 | 2.7.11.1 | CBM2 |
|  | lytic chitin monoxygenase | *lpmo* | HC2_GM001418 | 1477091:1477696 | 1.14.99.53 | AA10 |
|  | cellulose synthase |  | HC2_GM001470 | 1529207:1530250 | 2.4.1.12 | GT2 |
|  | cellulose synthase |  | HC2_GM001521 | 1579442:1581337 | 2.4.1.12 | GT2 |
|  | cellulose synthase |  | HC2_GM001526 | 1584704:1585984 | 2.4.1.12 | GT2 |
|  | cellulose synthase |  | HC2_GM001719 | 1790549:1791490 | 2.4.1.12 | GT2 |
|  | cellulose synthase |  | HC2_GM001742 | 1817027:1817977 | 2.4.1.12 | GT2 |
|  | cellulose synthase |  | HC2_GM001779 | 1856475:1857797 | 2.4.1.12 | GT2 |
|  | cellulose synthase |  | HC2_GM002270 | 2340747:2342075 | 2.4.1.12 | GT2 |
|  | cellulose synthase |  | HC2_GM002330 | 2398415:2399083 | 2.4.1.12 | GT2 |
|  | Cellulose biosynthesis protein BcsQ |  | HC2_GM002688 | 2775650:2776417 |  |  |
|  | cellulase activity |  | HC2_GM002734 | 2825055:2825597 |  |  |
| chitosan | chitosanase |  | HC2_GM003235 | Plas2:45849:46964 | 3.2.1.132 | GH8 |
| beta-glucan | exo-beta-1,4-glucanase |  | HC2_GM000378 | 392518:393954 | 3.2.1.74 | GH1 |
|  | endoglucanase |  | HC2_GM001009 | 1066049:1068268 | 3.2.1.4 | GH6 |
|  | exo-beta-1,4-glucanase |  | HC2_GM001118 | 1193686:1195158 | 3.2.1.74 | GH1 |
|  | exo-beta-1,4-glucanase |  | HC2_GM002001 | 2083803:2084768 | 3.2.1.74 | GH1 |
|  | exo-beta-1,4-glucanase |  | HC2_GM002325 | 2393496:2394938 | 3.2.1.74 | GH1 |
|  | exo-beta-1,4-glucanase |  | HC2_GM002326 | 2394956:2396377 | 3.2.1.74 | GH1 |
|  | exo-beta-1,4-glucanase |  | HC2_GM002528 | 2610795:2612177 | 3.2.1.74 | GH1 |
|  | exo-beta-1,4-glucanase |  | HC2_GM002632 | 2727982:2729415 | 3.2.1.74 | GH1 |
|  | endoglucanase E-like protein |  | HC2_GM002925 | 3003655:3004626 |  |  |
|  | exo-beta-1,4-glucanase |  | HC2_GM002992 | 3082443:3083903 | 3.2.1.74 | GH1 |
|  | exo-beta-1,4-glucanase |  | HC2_GM002995 | 3086987:3088435 | 3.2.1.74 | GH1 |
|  | exo-beta-1,4-glucanase |  | HC2_GM002996 | 3088524:3089987 | 3.2.1.74 | GH1 |
|  | exo-beta-1,4-glucanase |  | HC2_GM003084 | 3179552:3180463 | 3.2.1.74 | GH1 |
|  | Endoglucanase Y |  | HC2_GM003235 | Plas2:45849:46964 |  |  |

Note: At the column of Locus, Plas1 or Plas2 represent corresponding genes located on the plasmid 1 or plasmid 2, respectively. In addition, all other genes are located on the chromosome.

Table S4

The results of ANI (Average Nucleotide Identity) and DNA-DNA relatedness of strain HC-2 with [*Lactiplantibacillus plantarum*](https://www.ncbi.nlm.nih.gov/genome/1108) or *Lactiplantibacillus pentosus*

|  | HC-2 vs. [*L. plantarum*](https://www.ncbi.nlm.nih.gov/genome/1108) PC520 | HC-2 vs. *L. pentosus* SLC13 |
| --- | --- | --- |
| ANI: OrthoANIu value (%) | 99.45 | 80.15 |
| Probability that DDH > 70% (%)  (via logistic regression) | 97.21 | 0.12 |

**Note:** Probability that DDH > 70% was calculated based on formula 2 (identities / HSP length). DDH: DNA-DNA hybridization; HSP: high scoring pair.

**2 Supplementary Figures**


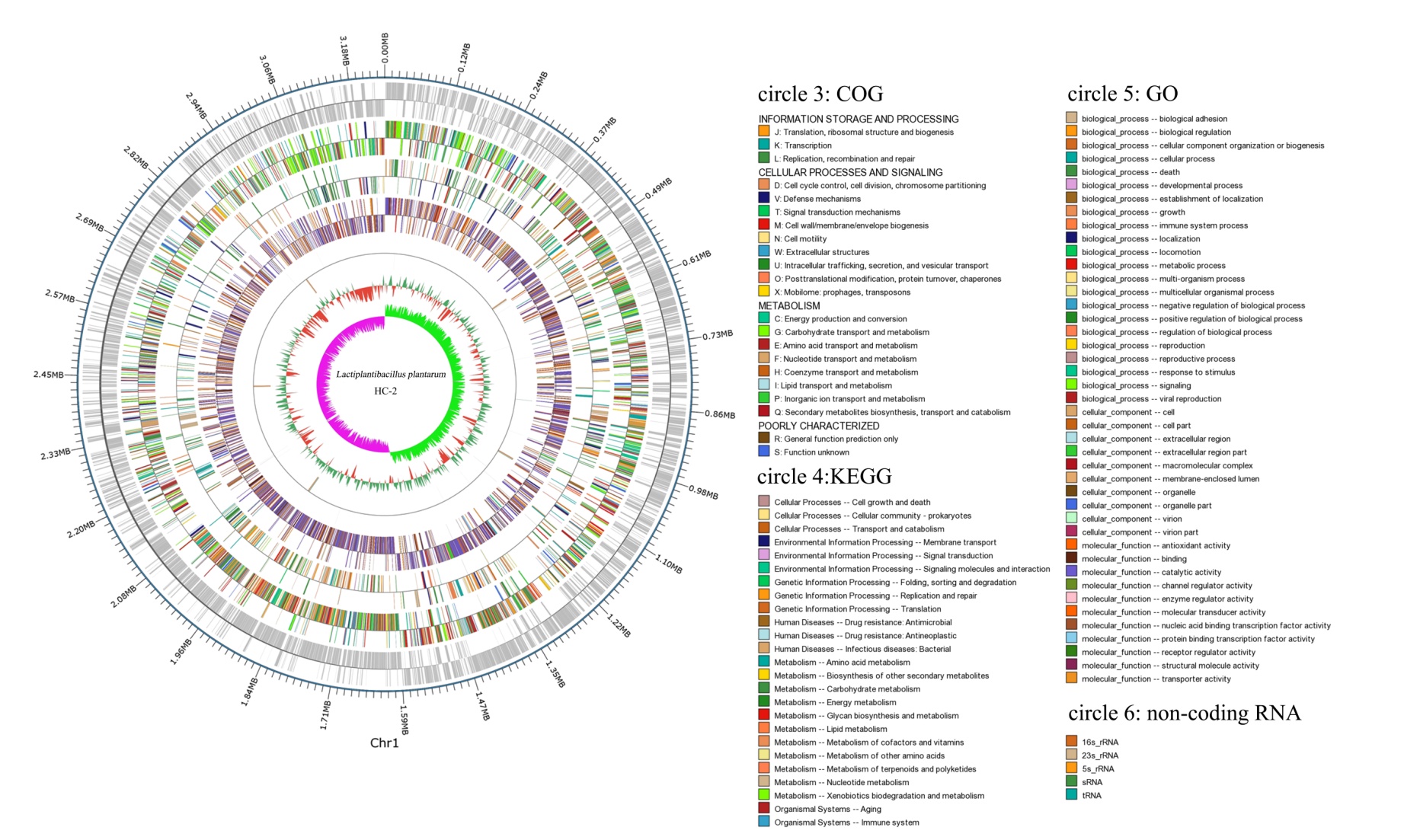


**Fig. S1 Chromosome structure of *Lactiplantibacillus plantarum* HC-2.** Circles were numbered from outside to inside. The outermost circle presents the coordinates, and the second circle depicts the predicted coding sequences. The third to fifth circles indicate the annotation of genes based on COG, KEGG and GO, respectively. The sixth circle depicts the non-coding RNA. The seventh circle indicates the GC content, the red part inward indicates that the GC content of the region is lower than that of the genome, while the dark green part outward is opposite, the higher peak means the greater difference between the GC content of the region with the average GC content of the genome. The eighth circle indicates the GC skew, the purple part inward indicates that the G content is lower than C content, while the green part outward is opposite.


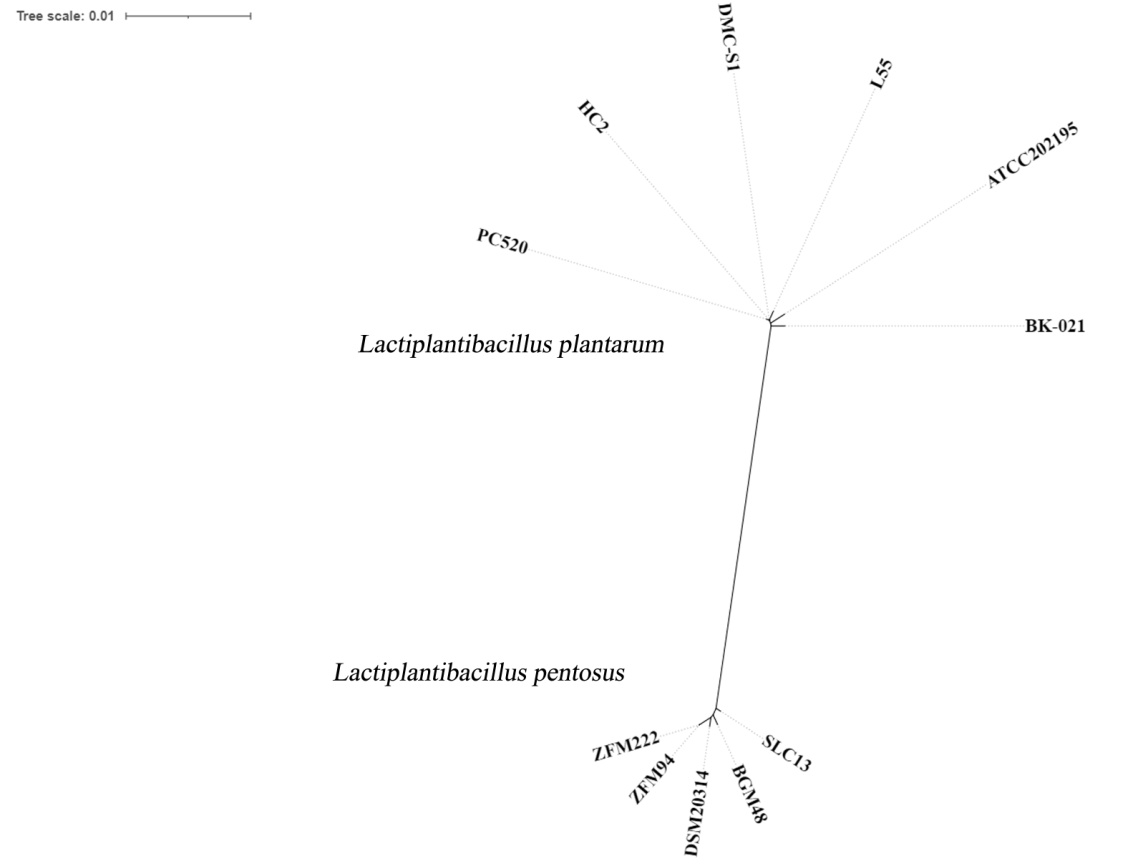


**Fig.S2 Phylogenetic relationship of strain HC-2 based on the assembly genome sequence.**
